# ACE: the Advanced Cohort Engine for searching longitudinal patient records

**DOI:** 10.1093/jamia/ocab027

**Published:** 2021-03-13

**Authors:** Alison Callahan, Vladimir Polony, José D Posada, Juan M Banda, Saurabh Gombar, Nigam H Shah

**Affiliations:** 1 Center for Biomedical Informatics Research, School of Medicine, School of Medicine, Stanford University, Stanford, California, USA; 2 Department of Computer Science, Georgia State University, Atlanta, Georgia, USA; 3 Department of Pathology, School of Medicine, Stanford University, Stanford, California, USA

**Keywords:** electronic health records, in-memory datastore, query language, search engine, data science

## Abstract

**Objective:**

To propose a paradigm for a scalable time-aware clinical data search, and to describe the design, implementation and use of a search engine realizing this paradigm.

**Materials and Methods:**

The Advanced Cohort Engine (ACE) uses a temporal query language and in-memory datastore of patient objects to provide a fast, scalable, and expressive time-aware search. ACE accepts data in the Observational Medicine Outcomes Partnership Common Data Model, and is configurable to balance performance with compute cost. ACE’s temporal query language supports automatic query expansion using clinical knowledge graphs. The ACE API can be used with R, Python, Java, HTTP, and a Web UI.

**Results:**

ACE offers an expressive query language for complex temporal search across many clinical data types with multiple output options. ACE enables electronic phenotyping and cohort-building with subsecond response times in searching the data of millions of patients for a variety of use cases.

**Discussion:**

ACE enables fast, time-aware search using a patient object-centric datastore, thereby overcoming many technical and design shortcomings of relational algebra-based querying. Integrating electronic phenotype development with cohort-building enables a variety of high-value uses for a learning health system. Tradeoffs include the need to learn a new query language and the technical setup burden.

**Conclusion:**

ACE is a tool that combines a unique query language for time-aware search of longitudinal patient records with a patient object datastore for rapid electronic phenotyping, cohort extraction, and exploratory data analyses.

## INTRODUCTION

In a learning health system, the capability to search large collections of patient records is essential.^1–3^ Many tools have been developed for searching patient data that are designed for a range of use cases including data entry and retrieval to inform clinical care,^4–7^ identifying patient cohorts from clinical data warehouses for research studies,^8–14^ securely federating search across clinical data warehouses,^15^ data delivery,^16^ creating clinical dashboards,^17^ exploring clinical text,^18^ and “always on” alerting systems that flag patients for potential enrollment in clinical trials based on electronic medical records in health systems.^19^^,^^20^

Querying patient data typically relies on a form-based interface^13^^,^^16^^,^^21^^,^^22^ which allows specifying a cohort of interest by selecting 1 or more features from a list of possible features, using elements such as text boxes and dropdown lists of possible values to specify the restrictions on features. Such form-based modes of interaction are in contrast to *search* which dominates content access on the World Wide Web. Search minimizes the structured elements required to initiate a search and emphasizes speed and scalability. These 2 approaches—of structured query *versus* search—have different trade-offs: form-based interfaces enable complex and detailed queries with high precision, but require a high level of user expertise; search engines prioritize simplicity at the expense of ambiguity in the meaning of searches, potentially resulting in a mismatch between the intent of a given search and its results.

To combine the ease of search with the expressivity and precision of structured queries, we developed the Advanced Cohort Engine (ACE).^23^ Central to ACE is a temporal query language (TQL) that operates over an in-memory datastore of patient objects in which all the data of a single patient are stored together and indexed both by a precomputed patient feature index and by the time they occurred. This design avoids the performance issues that arise in relational databases,^24^ which have no inherent notion of time and rely on computationally expensive join operations to query across record types and sequences of events. As an example of the kind of search ACE enables, consider a scenario in which a user wishes to find type II diabetic patients for whom first line therapy proved ineffective.^25^ They have to identify type II diabetic patients who received a first line diabetes medication *for the first time* after their diagnosis, and then restrict this group to those whose glycated hemoglobin (HbA1c) remained high *after* that medication appeared in their record. Doing so requires the ability to query diagnosis records, medication records, and laboratory test results and to express complex temporal relationships between them including *before*, *after,* and *for the first time*. In addition, this task requires specifying what “diabetes medications” are and which ones qualify as “first line.” ACE can traverse multiple knowledge graphs during search for this purpose, to retrieve patient records of any *drug used to treat diabetes* via parent-child and used-to-treat relationships from public ontologies.

ACE enables a conversational approach for interacting with data, wherein a user initiates a search to get a response within a few seconds and can inspect the results retrieved to validate the search criteria. This is especially useful for electronic phenotyping, the process of defining the necessary and sufficient criteria for identifying patients with a condition of interest. Moreover, the “conversation” between the user and dataset, which ACE facilitates, enables rapid iteration, so that users can quickly devise new searches based on the results of prior searches, essentially combining electronic phenotyping and cohort-building into a single iterative search process. This combined functionality supports high-value use cases that health systems and academic medical centers have, such as data vending, quality metric reporting, rapid clinical trial recruitment, generating labeled data for machine learning, and using aggregate patient data at the bedside.

In the remainder of the article, we describe the design and architecture of ACE, the TQL, and the user interface. We describe detailed use cases for ACE with example searches, and present the results of experiments to evaluate ACE performance. Lastly, we discuss the benefits and limitations of ACE.

## OBJECTIVE

The objective of this work is to propose a new paradigm for scalable search of longitudinal patient records via an expressive TQL coupled with an in-memory patient datastore and to describe the implementation, use cases, and performance of a search engine realizing this paradigm.

## MATERIALS AND METHODS

The existing industry standard in cohort-building tools is a combination of a relational database back end with a front end to translate form-generated queries from a user interface into SQL queries. Solutions that assist in query formulation via a form-based visual query UI (such as ATLAS by the observational data science and informatics [OHDSI] community^26^) determine the time from question ideation to query formulation. Query execution time and result retrieval time are based largely on the performance of the underlying database engine.

A conversational approach to interacting with data requires the time from question ideation to query formulation as well as the subsequent query execution and result retrieval to be extremely short. We first describe the TQL and query execution process that enables such rapid search followed by how ACE uses knowledge graphs to expand search results as well as details of the in-memory datastore search and the different retrieval mechanisms. We then describe the 2 user interfaces for search and result inspection. ACE is designed to be compatible with OHDSI and the Observational Medical Outcome Partnership (OMOP) Common Data Model (CDM). Details of the ACE extract-transform-load (ETL) from the OMOP CDM are provided in the Supplementary Materials, along with details on how to license ACE for commercial or (free) academic use. ACE can operate over data structured using other schemas provided they contain at least 1 of the search elements that ACE supports (eg, ICD codes or medication records). ACE can also be used with other clinical CDMs such as i2b2 via custom ETLs. We have tested this functionality by developing custom ETLs for other source databases, such as IBM MarketScan and the Optum Clinformatics Data Mart.

### The temporal query language

The Structured Query Language (SQL) underlying most relational databases does not natively support expressing temporal relationships among data elements or executing temporal queries. Searching by timing of events in a patient’s longitudinal record with SQL is dependent on the table structures that capture information about when in time a clinical event occurred. Prior efforts have either focused on developing novel temporal language commands extending the SQL language itself and implemented in the underlying SQL engine^27^ or on developing interfaces that allow translating a temporal query into a SQL query.^26^^,^^28–32^ Our effort focused on developing a novel TQL implemented in a non-SQL datastore.^33^^,^^34^

The ACE TQL introduces a temporal algebra which allows expression of complex temporal relationships in a human readable way that is agnostic of the source data structure. Schema agnosticism is necessary to allow consistent querying over multiple datasets potentially structured using multiple schemas. Therefore, we abstract the language algebra from the features seen in individual datasets. TQL thus consists of 2 components: an *immutable language algebra* and *feature-specific commands*. Feature-specific commands depend on the source data. In the case of electronic health records and insurance claims data, the features include diagnosis and procedure codes (ICD9, ICD10, CPT), drug codes (RxCUI and ATC), visit types, note types, mentions of clinical terms in notes, encounters, insurance plan enrollment information, laboratory and vitals measurements, ages, years, and demographics. To search clinical notes content, the TEXT command allows users to specify a word or phrase to search for as well as modifiers (including the kind of note, whether the word/phrase is negated, and whether the word/phrase occurs in the context of family history). The set of possible words and phrases that can be searched depends on the text processing system used to generate the processed data that ACE ingests during ETL, such as Trove^35^ (a system we developed for concept and relation extraction that is OMOP CDM compatible), cTAKES,^36^ and MedLEE,^37^ among many others. ACE’s feature-specific commands can be modified for different needs since they exist separately from the language algebra.

The TQL algebra encapsulates the Boolean and temporal relationships that can be expressed in TQL. TQL’s Boolean algebra consists of AND (return patients with a record of *all* specified features at any time), OR (return patients with a record of *any* of the specified features at any time), and NOT (return patients *without any* of the specified features). The corresponding temporal algebra consists of INTERSECT (return time intervals where it was true that a patient had a record of *all* specified features *occurring at the same time*), UNION (return the time intervals where it was true that a patient had a record of *any* of the specified features *occurring at any time*), INVERT (return the time intervals where it was *not* true that a patient had a record of *any* of the specified features) and SEQUENCE (return time intervals if 2 events happen in a particular time sequence). TQL supports over 100 different commands, many of which are built upon these core commands, providing high expressivity in temporal queries. As an example, consider the following cohort definition: *Male patients over 65 years old who have type II diabetes (defined by at least 2 occurrences of type II diabetes ICD9 codes or 2 elevated A1C lab results) with no history of stroke and who went on to have a stroke within 3 months after administration of glipizide.*

This cohort definition is shown as TQL commands in Box 1. The first few lines define what we mean by **stroke**, **patients older than 65 years,** and **glipizide**, each item becoming their own variable. A variable definition starts with the keyword **var** followed by the name of the variable, which is followed by the TQL expression defining the variable. The variables can then be used in subsequent variables, or as a query, by referencing their name preceded by the $ character.

- **Stroke** is defined as **ICD9** code 434.91
- **Male patients over 65** is an intersection of people with male **GENDER** and with **AGE** over 65.
- **Glipizide** is defined as **RX** 310490 (RX codes are drawn from RxNorm’s RxCUI set). We could expand the definition to other medications of the same class (glucose-lowering drugs) by using mappings from RxCUIs to the Anatomic Therapeutic Code (ATC) classification system.
- **No history of stroke** uses the **NO HISTORY OF** command to return either the entire patient’s timeline (if stroke never occurred for a patient), or the part of the timeline before any stroke occurred.
- **Type II diabetes** is a bit more complicated. It takes patients who had at least 2 instances of ICD9=“250.00” (the ICD9 code for type II diabetes mellitus) or 2 instances of high HbA1c measurement (indicated by the LOINC code 4548-8). The **UNION** command takes the combination of both of these, and **FIRST MENTION** returns the earliest of the intervals returned by the **UNION**, to determine the *start* of each patient’s diabetes diagnosis.
- **Diabetes with no history of stroke** uses **INTERSECT** to return the temporal intersection of the first occurrence of diabetes with the portion of each patient’s record where they had not experienced a stroke event. This query will exclude patients who had a stroke prior to their diabetes diagnosis.
- **Diabetes then glipizide** is a **SEQUENCE** command that looks for patients with an occurrence of diabetes (with no history of stroke) followed by a prescription of glipizide. The “*” modifier indicates which time intervals to return. In this case, we want the glipizide time intervals to be returned, so they can be used to restrict the search for subsequent stroke events.
- **Glipizide then stroke** takes the result of the previous variable and looks for a stroke in the following 3 months. The **SEQUENCE** command is very versatile and can be followed by parameters to, for example, specify the presence or absence of events within a given time range (see the ACE TQL documentation in the Supplementary Materials for a full description of the SEQUENCE command syntax).

The final command, executed as a query, looks for the intersection of stroke events following glipizide prescriptions in male patients over the age of 65.

Box 1. Temporal query language commands to define a cohort of male patients over 65 years old who are type II diabetic, with no history of stroke, and who went on to have a stroke within 3 months after administration of glipizide

varstroke= ICD9=“434.91”

varmale_patients_over_65 = INTERSECT(GENDER=”male”, AGE(65 YEARS, MAX))

varglipizide= RX = 310490

varno_history_of_stroke= NO HISTORY OF ($stroke)

vardiabetes= FIRST MENTION(UNION(COUNT(ICD9=“250.00”, 2, MAX),

COUNT(LABS(“4548-4 [%]”, 8, MAX), 2, MAX)))

vardiabetes_no_hx_stroke= INTERSECT($diabetes,$no_history_of_stroke)

vardiabetes_then_glipizide= SEQUENCE($diabetes_no_hx_stroke,$glipizide*)

varglipizide_then_stroke= SEQUENCE($diabetes_then_glipizide,$stroke*)+(-3 MONTHS, 0)

INTERSECT($male_patients_over_65,$glipizide_then_stroke)

### Use of knowledge graphs in specific commands

For features such as ICD9 and 10 codes, which have a hierarchy among them, ACE stores the hierarchical expansion resulting from a transitive closure on the parent-child relationship in the in-memory patient object^38^^,^^39^ motivated by prior work on incorporating ontology-derived knowledge into database querying examples.^40–45^ If a child node exists in a patient’s data, a query for any of its parent nodes will return that patient record. For example, if ICD9=“250.02” exists in a patient's timeline, then during the extraction process, all the parent nodes of the “250.02” code are also associated to the same time point in the patient object. Querying ICD9=“250.0” and ICD9=“250”, as well as ICD9=“250.02” will return the patient record. When such hierarchical expansion is not desired, it can be turned off by using the ORIGINAL command, which returns only the codes that were originally present in the source data.

Hierarchical expansion is available for ICD9 and ICD10 codes as well as for drugs via the Anatomic Therapeutic Chemical (ATC) classification system hierarchy. Every drug’s RxNorm^46^ identifier (RxCUI) is expanded to the appropriate ATC parent nodes based on mappings between RxCUIs and ATC codes and ATC child nodes to their parent nodes. For example, patient records for acetaminophen (RxCUI 161) will include the following ATC classes:

- N02BE (Anilides)
- N02B (OTHER ANALGESICS AND ANTIPYRETICS)
- N02 (Analgesics)
- N (Nervous System)

This means that instead of having to list all analgesics in a query, we can use the ATC class (N02) to find patients who received an analgesic.

### Fast search over a datastore of patient objects

In the ACE search engine, instead of grouping and storing data into tables by feature (for example, all ICD diagnosis codes in 1 table, all laboratory test results in another, with additional dictionary tables for ICD codes and laboratory test codes), ACE data are grouped and stored as patient objects (one per patient record), and organized by a feature index.^47–49^ As a result, it is possible to perform very fast lookup operations using single features to determine which patient objects need to be further evaluated to determine whether they should be included in the result of a search. A full evaluation—examining all features against all declared search constraints—is done on only the subset of patient objects that have a chance to produce a positive result. This second evaluation is very fast since all the data for a given patient can be inspected without having to perform additional queries or table joins.

Consider the example of identifying patients over 65 with diabetes and high blood glucose from the 2.8 million Stanford Health Care patients in our local ACE instance. ACE fully evaluates only the patients with the following features:

1. Male patients over 65 years old (∼270 000 patients)
2. Patients with ICD9 250.00 (∼58 000 patients)
3. Patients with a blood glucose lab (∼260 000 patients)

Fewer than 21 000 patient records have all 3 of these features. The operation to determine the number of patients to be fully evaluated further to decide if they are a valid match to the query takes 38 milliseconds—far less time than sequentially evaluating 2.8 million patient records to find the 58 000 with the code 250.00 and then sequentially determining the overlap with those that are over 65 and have a blood glucose lab.

This setup overcomes the main limitation of relational databases where data are separated into specific tables and wherein a search for a specific feature value requires examining the entire table. While indexing certainly speeds up this process, increasing the size of the table decreases performance; this makes it difficult to scale up without a substantial increase in resource usage. In addition, as features are added to a query, it requires a search in other tables. In the worst-case scenario (a search criteria that includes every feature type such as diagnosis codes, drugs, age, demographics, procedure codes, laboratory results, etc), the search must touch every table in the database. Relational databases also rely on dictionaries, where a data table contains values that are mapped to 1 or more other tables (a dictionary), which contains a record for each unique feature value. While this decreases the size of the primary data table, a query requires a JOIN operation with the dictionary table(s) to execute a search. This process is computationally expensive and, in the case of large data tables and large dictionary tables, can result in queries taking days to complete.

### Scalability with distributed computing

The ACE datastore is separated into shards, which can be partially or fully loaded into memory. Optimal performance is achieved by loading all the shards into the memory, which leads to query times of less than 1 second. Increasing the number of shards read from disk reduces the hardware requirements but leads to query performance degradation. Shards are autonomous and self-contained such that the entire patient datastore can be split into different subsets, which can be then instantiated on different computers. One computer then functions as a primary node that distributes queries into other secondary nodes in a cluster, which can further speed up query execution performance. In the results, we present 2 server configurations and the average query times these configurations achieve for core TQL commands.

### ACE user interfaces

**Figure 1.**
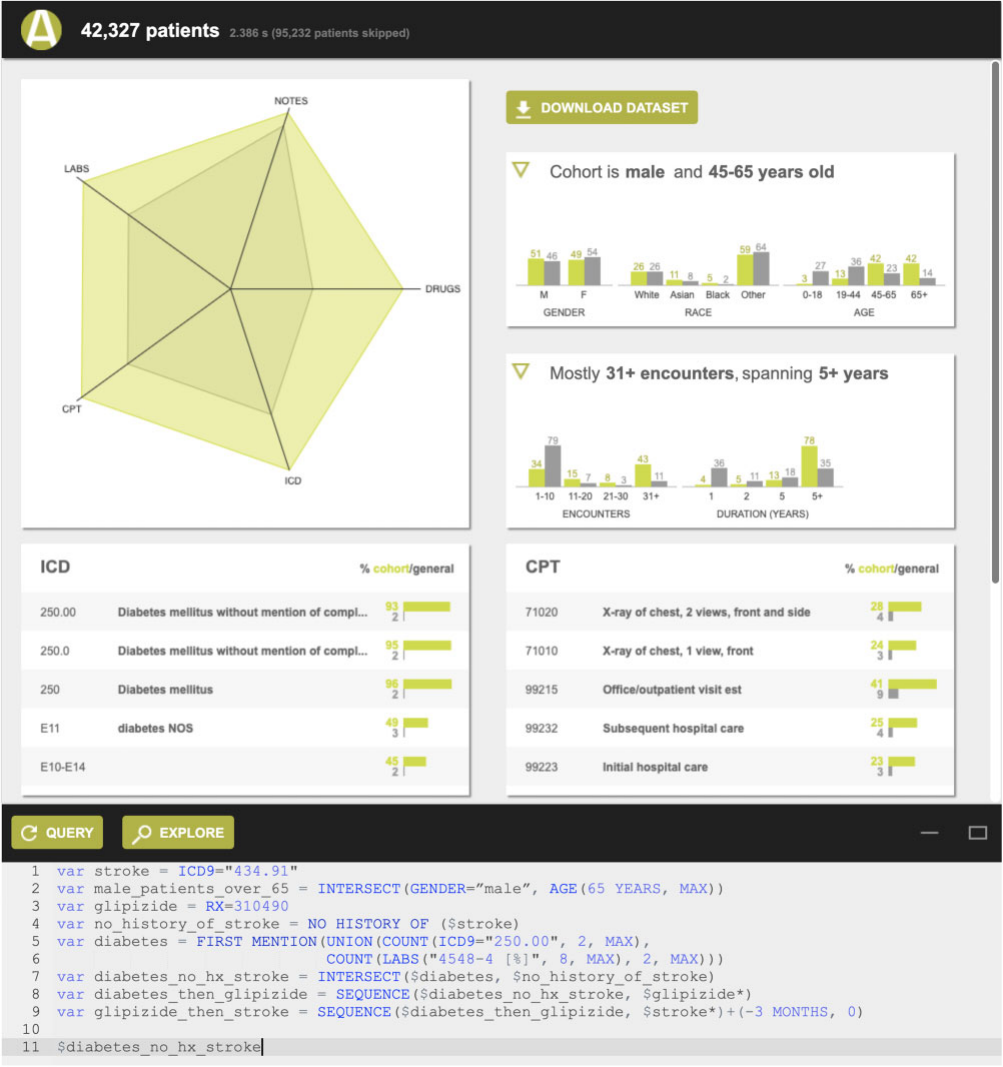
Summary view showing the number of patients meeting the search criteria, a summary of their demographics (histograms of age, race/ethnicity, and length of record), and their most frequently occurring diagnosis, procedure, medication, and laboratory test records.

**Figure 2.**
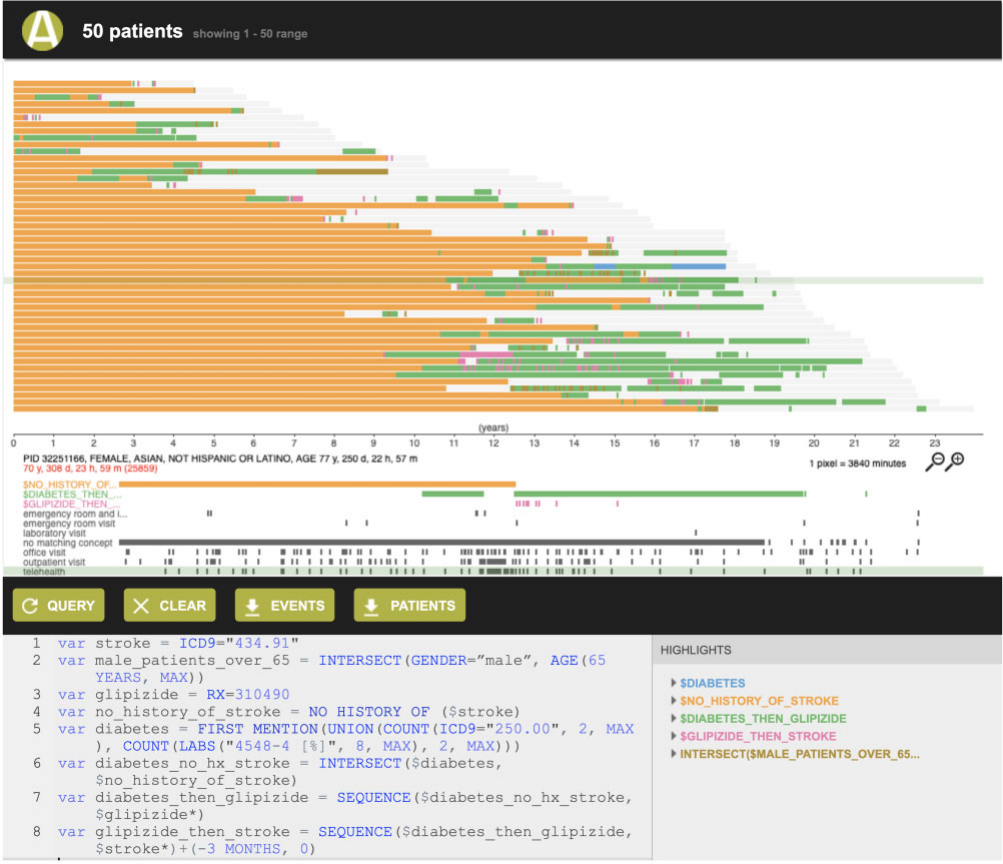
Patient timeline view, displaying each patient as a row and showing the time intervals where a given search criterion was satisfied in different colors. For example, glipizide prescription records following type II diabetes diagnosis are shown in green, and subsequent stroke events are shown in pink.

ACE shows search results in 2 ways: (1) a **summary view** of patient records (Figure 1) retrieved by a search, which includes the number of patient records meeting the search criteria, a summary of the demographics (histograms of age, race/ethnicity, and length of record), and the most frequently occurring diagnosis, procedure, medication, and laboratory test records; and (2) a **patient timeline view** (Figure 2) that represents each patient record retrieved as a horizontal timeline, highlighting the time(s) that a given search criterion was true. The patient timeline view, motivated by prior work demonstrating the value of such visualization,^18^^,^^50–56^ allows a user to rapidly inspect search results to determine if they are what the user intended to retrieve.

### Data retrieval

ACE is a representational state transfer (REST) application programming interface (API) search engine, so its functionality is available both via a Web UI and by directly querying the underlying API. We have also developed Java, Python, and R libraries for calls to the ACE API. These libraries also provide the ability to convert the JSON responses from ACE API calls into data objects that can be directly used with these programming languages. Complete API documentation is available in the Supplementary Materials. The API code is available at https://github.com/som-shahlab/ACEapi and the R package at https://cran.r-project.org/web/packages/ACEsearch/.

## RESULTS

ACE’s unique combination of a TQL for expressing complex temporal relationships among data elements with fast retrieval enabled by an in-memory datastore of patient objects supports a variety of use cases. We describe these, along with example queries. We then report on experiments to profile ACE’s performance and provide a comparative summary with existing search tools for clinical data in the Supplementary Materials.

### Use cases

Electronic phenotyping is the process of defining the necessary and sufficient criteria that a patient’s record must satisfy to consider an exposure or outcome to have occurred for that patient.^57^ Combining the process of defining the phenotype with the ability to retrieve and inspect the patient records that satisfy those criteria in a patient timeline view allows real-time review and rapid iteration of phenotype definitions, enabling multiple use cases.

#### Using aggregate patient data at the bedside

The first use of ACE was to support the vision of using aggregate patient data at the bedside^2^ via an informatics consultation service, which was an IRB-approved study of the use of routinely collected data on millions of individuals to provide on-demand evidence in situations where good evidence is lacking.^58^ ACE was used to determine the size of patient cohorts meeting a set of specific criteria and to retrieve the patient records for subsequent statistical analyses. These tasks subsume activities referred to as electronic phenotyping, or a cohort definition if sufficiently complex, and cohort retrieval.^59^

Over the course of 1.5 years, ACE was used to define an estimated 5000 electronic phenotypes and retrieve the associated patient cohorts. Offering consultations thus served as a functional assessment of the utility of ACE and its ability to address the cohort discovery and creation needs of users. The criteria expressed ranged in complexity from single 1-line queries (eg, patients who underwent spinal surgeries, as defined by a series of CPT codes) to queries that combined many commands (eg, adult patients with a diagnosis of diffuse B-cell lymphoma who received Neulasta, but not Neupogen, as well as chemotherapy within the 3 days preceding or 8 days following Neulasta administration, and then went on to have neutropenia, defined as an absolute neutrophil count less than 500 within 28 days of the start of their chemotherapy regimen).

ACE made it possible to iterate on complex query definitions, in partnership with clinicians, until the desired patient cohort was retrieved for each consultation. To assess feasibility, often a derivation of simple summary statistics (eg, the mean of a value) was required, which was enabled by data export functions allowing patient-level retrieval of laboratory test results and other numeric values (eg, height, weight, body temperature). Being a clinician-facing service, consultations had to be completed within 1–3 days, which was only possible by reducing the time spent on phenotyping and cohort-building to a few hours, allowing sufficient time to be spent on statistical analyses required by the consultation request.

Since then, a number of other use cases have emerged, all powered by the ability to combine electronic phenotyping and cohort-building into a rapid, unified, and iterative process.

#### Quality metric reporting

Hospital operations teams require mechanisms to generate reports summarizing hospital performance along many axes, including patient outcomes. Many such performance indicators are quantified using quality metrics developed and maintained by independent entities such as the National Committee for Quality Assurance (NCQA). Quality metrics require the ability to identify patients with a given diagnosis or who received a specific therapy, and to track subsequent outcomes including readmission, infection, or death.

Each of these criteria are essentially an electronic phenotype and are thus amenable for execution using ACE. For example, we can translate NCQA Healthcare Effectiveness Data and Materials Set (HEDIS) measures to ACE queries (see Box 2 for an example), which can then be incorporated into performance reports.

Box 2. ACE variables for HEDIS measure on the avoidance of antibiotics to treat bronchitis

varage= AGE(18 YEARS, 64 YEARS)

varbronchitis= ICD9=“466.0”

varhiv= ICD9=“042”

varmal_neo= UNION(ICD9=“140”, ICD9=“141”, ICD9=“142”, ICD9=“143”, ICD9=“144”, ICD9=“145”, ICD9=“146”, ICD9=“147”, ICD9=“148”, ICD9=“149”, ICD9=“150”, ICD9=“151”, ICD9=“152”, ICD9=“153”, ICD9=“154”, ICD9=“155”, ICD9=“156”, ICD9=“157”, ICD9=“158”, ICD9=“159”, ICD9=“160”, ICD9=“161”, ICD9=“162”, ICD9=“163”, ICD9=“164”, ICD9=“165”, ICD9=“170”, ICD9=“171”, ICD9=“172”, ICD9=“173”, ICD9=“174”, ICD9=“176”, ICD9=“180”, ICD9=“182”, ICD9=“183”, ICD9=“184”, ICD9=“186”, ICD9=“187”, ICD9=“188”, ICD9=“189”, ICD9=“190”, ICD9=“191”, ICD9=“192”, ICD9=“194”, ICD9=“195”, ICD9=“196”, ICD9=“197”, ICD9=“198”, ICD9=“199”, ICD9=“200”, ICD9=“201”, ICD9=“202”, ICD9=“203”, ICD9=“204”, ICD9=“205”, ICD9=“206”, ICD9=“207”, ICD9=“208”, ICD9=“209”)

varemphysema= UNION(ICD9=“492”)

varcopd= UNION(ICD9=“493.2”, ICD9=“496”)

varcystic_fibrosis= UNION(ICD9=“277.0”)

varccvs= UNION(ICD9=“279”, ICD9=“491”, ICD9=“494”, ICD9=“495”, ICD9=“500”, ICD9=“506”, ICD9=“507”, ICD9=“508”, ICD9=“510”, ICD9=“511”, ICD9=“512”, ICD9=“513”, ICD9=“516”, ICD9=“517”, ICD9=“518”, ICD9=“519”, ICD9=“010”, ICD9=“011”, ICD9=“012”, ICD9=“013”, ICD9=“014”, ICD9=“015”, ICD9=“016”, ICD9=“017”, ICD9=“018”)

varcdvs= UNION(ICD9=“001”, ICD9=“002”, ICD9=“003”, ICD9=“004”, ICD9=“005”, ICD9=“006”, ICD9=“007”, ICD9=“008”, ICD9=“009”, ICD9=“033”, ICD9=“041.9”, ICD9=“088”, ICD9=“382”, ICD9=“461”, ICD9=“462”, ICD9=“034.0”, ICD9=“473”, ICD9=“464.1”, ICD9=“464.2”, ICD9=“464.3”, ICD9=“474”, ICD9=“478.21”, ICD9=“478.24”, ICD9=“478.29”, ICD9=“478.71”, ICD9=“478.79”, ICD9=“478.9”, ICD9=“601”, ICD9=“383”, ICD9=“681”, ICD9=“682”, ICD9=“730”, ICD9=“686”, ICD9=“482”, ICD9=“483”, ICD9=“484”, ICD9=“486”, ICD9=“098”, ICD9=“099”, ICD9=“V01.6”, ICD9=“090”, ICD9=“091”, ICD9=“092”, ICD9=“093”, ICD9=“094”, ICD9=“095”, ICD9=“096”,ICD9=“097”, ICD9=“098”, ICD9=“099”, ICD9=“078.88”, ICD9=“079.88”)

varantibiotic= UNION(RX = 641, RX = 142438, RX = 10109, RX = 10627, RX = 723,RX = 733, RX = 8339, RX = 10591, RX = 2177, RX = 2180, RX = 2231, RX = 20481, RX = 274786, RX = 2582, RX = 18631, RX = 21212, RX = 4053, RX = 1272, RX = 2348, RX = 229369, RX = 22299, RX = 190376, RX = 6922, RX = 11124, RX = 7980, RX = 7984, RX = 3356, RX = 7233, RX = 7773, RX = 9384, RX = 2176, RX = 2187, RX = 19552, RX = 2189, RX = 2194, RX = 10171, RX = 10180, RX = 3640, RX = 6980, RX = 10395, RX = 25037, RX = 83682, RX = 25033, RX = 2186, RX = 20489, RX = 2191, RX = 20492, RX = 2193, RX = 4550, RX = 7454, RX = 10829)

varbronchitis_cohort= INTERSECT($bronchitis,$age)

varno_ccvs= UNION(INTERSECT($bronchitis_cohort, NEVER HAD($ccvs)),SEQUENCE($ccvs,$bronchitis_cohort*)-(-1 YEAR, 1 DAY))

varno_cdvs= UNION(INTERSECT($bronchitis_cohort, NEVER HAD($cdvs)),SEQUENCE($cdvs,$bronchitis_cohort*)-(-30 DAYS, 8 DAYS))

varno_emphysema= UNION(INTERSECT($bronchitis_cohort, NEVER HAD($emphysema)), SEQUENCE($emphysema,$bronchitis_cohort*)-(-1 YEAR, 1 DAY))

varno_copd= UNION(INTERSECT($bronchitis_cohort, NEVER HAD($copd)), SEQUENCE($copd,$bronchitis_cohort*)-(-1 YEAR, 1 DAY))

varno_cf= UNION(INTERSECT($bronchitis_cohort, NEVER HAD($cystic_fibrosis)), SEQUENCE($cystic_fibrosis,$bronchitis_cohort*)-(-1 YEAR, 1 DAY))

varno_hiv= UNION(INTERSECT($bronchitis_cohort, NEVER HAD($hiv)), SEQUENCE($hiv,$bronchitis_cohort*)-(-1 YEAR, 1 DAY))

varno_mal_neo= UNION(INTERSECT($bronchitis_cohort, NEVER HAD($mal_neo)), SEQUENCE($mal_neo,$bronchitis_cohort*)-(-1 YEAR, 1 DAY))

varno_antibiotic= UNION(INTERSECT($bronchitis_cohort, NEVER HAD($antibiotic)), SEQUENCE($antibiotic,$bronchitis_cohort*)-(-1 MONTH, -1 DAY))

vardenominator= INTERSECT($no_ccvs,$no_cdvs,$no_emphysema,$no_copd,$no_cf,$no_hiv,$no_mal_neo,$no_antibiotic)

varbronchitis_then_antibiotic= SEQUENCE($denominator,$antibiotic*) + (-3 DAYS, 0 DAYS)

varnumerator= DIFF($denominator,$bronchitis_then_antibiotic)

$numerator

#### Clinical trial recruitment

The first step in clinical trial recruitment involves using inclusion and exclusion criteria for participants for identifying eligible patients who meet those criteria. Trial managers need the ability to search patient records using the clinical trial criteria to identify potential eligible participants or to set up automatic alerting when a patient meeting a given trial’s criteria receives care from their health system,^60^^,^^61^ and a great number of systems have been previously developed to support eligibility screening.^62^^,^^63^

ACE allows the expression of clinical trial inclusion and exclusion criteria as variables, which can be executed as a 1-time search to estimate the eligible patient pool, or on an ongoing basis via the ACE API to monitor for newly matched records. For example, Box 3 shows inclusion criteria for the Elevate! Clinical Trial as an ACE search.

Box 3. Inclusion criteria to identify candidate participants for the Elevate! clinical trial.

varage= AGE(70 YEARS, MAX)

varfemale= GENDER=“FEMALE”

varbcis= INTERSECT(ICD9=“233.0”, NO HISTORY OF(ICD9=“174”))

INTERSECT($bcis,$age,$female)

#### Generating labeled training data

There is a growing body of research on the benefits and tradeoffs of using “imperfectly labeled” data to train machine learning models.^64^^,^^65^ This research has demonstrated that when data can be computationally labeled at a scale sufficient for deep learning, the performance gains of models learned using large training data sets often outweigh potential inaccuracies in automated labeling methods, in comparison to models trained on smaller, expert-labeled data (which are expensive to obtain at a large scale).^66–68^ For example, we developed a weakly supervised method to identify complications of post-implant complications in hip replacement patients,^69^ including the time that a complication occurred. Assigning the time of events is essential when creating labeled training data for such use cases.

ACE enables the generation of large labeled training data sets by combining its TQL, which expresses complex electronic phenotypes that can determine the time that a phenotype was true for each patient, with the API that outputs data as flat files. For example, if we define type 2 diabetes (T2D) as anyone with at least 2 T2D codes or 2 occurrences of an abnormal blood glucose laboratory test result, and no type 1 diabetes diagnosis codes, the ACE query in Box 4 would retrieve those patients satisfying that definition, as well as the *time* that definition was true, allowing us to use these data to train a model to predict onset of T2D.

Box 4. Labeling diabetic patients and the time that they first met the necessary criteria.

vart2d= FIRST MENTION(UNION(COUNT(ICD9=“250.00”, 2, MAX), COUNT(LABS(“4548-4 [%]”, 8, MAX), 2, MAX)))

vart1d= OR(ICD9=“250.01”, ICD9=“250.03”)

vart2d_no_t1d= INTERSECT($t2d, NOT($t1d))

EXPORT($t2d_no_t1d, TIME=$t2d_no_t1d, “T2D”=$t2d_no_t1d)

#### Institution wide data vending services

Hospitals and schools of medicine often rely on dedicated teams of data analysts and scientists to provide datasets for research, clinical care, and operational purposes. The core work of these teams, often called “honest broker” teams,^70^ involves searching, retrieving, and manipulating patient data records that meet 1 or more electronic phenotype definitions at specific time points. As a single tool that enables temporal querying, phenotyping, and cohort extraction in various data output formats, ACE is well suited to support institution wide cohort extraction services for accelerating efforts supporting clinical data science.^71^

### Performance

We measured the time needed to define the type II diabetes cohort in Box 1 using ACE’s TQL and the time required to define the cohort using SQL over an OMOP CDM v5.3 BigQuery database containing the same data. It took under 5 minutes to define the type II diabetes cohort in TQL. The same cohort expressed in SQL took an experienced research engineer 2.5 hours to construct. The TQL query is 9 lines, while the corresponding SQL query is over 240 lines (see Supplementary Materials, Query writing times usingTQL and BigQuery SQL on OMOP CDM).

To quantify the performance of ACE on average, we generated 100 queries for the 5 core commands (AND, OR, INTERSECT, UNION, SEQUENCE), where the query parameters were randomly specified as any of ICD9, ICD10, CPT, or RX, or some combination thereof. We executed these queries on an ACE datastore composed of records from Stanford Medicine’s clinical data warehouse (CDW) containing data for 2.8 million patients, deployed on a single computer with 2 cores and 37 GB of RAM. We also executed equivalent SQL queries on our School of Medicine BigQuery instance over the same Stanford Medicine CDW records. Lastly, we executed these queries on an ACE datastore composed of insurance claims records for ∼65 million patients from the Optum Clinformatics Datamart, deployed on a cluster of 3 computers with 4 cores each and 127 GB of RAM. The query execution times resulting from these experiments are summarized in Table 1.

**Table 1.** Average query execution times in seconds for 100 randomly generated queries over electronic health records for ∼2.8 million patients from the Stanford Medicine CDW, using ACE or BigQuery; and over health insurance claims records for ∼65 million patients from the Optum Clinformatics Datamart, using ACE

Command	Average [min-max] query response time (seconds)		
	Stanford Medicine CDW		Claims
	ACE	BigQuery	ACE
**OR of 4 features**	0.015 [0.005–0.211]	198.7 [121.0–642.0]	1.224 [0.017–1.813]
**UNION of 4 features**	0.018 [0.005–0.306]	167.7 [73.0–227.0]	0.205 [0.026–1.618]
**AND of 2 features**	0.026 [0.005–0.681]	221.5 [124.0–940.0]	0.0684 [0.026–1.530]
**INTERSECT of 2 features**	0.024 [0.005–0.314]	233.6 [152.0–748.0]	1.018 [0.023–0.545]
**SEQUENCE of 2 features**	0.017 [0.005–0.214]	202.3 [148.0–266.0]	0.0602 [0.018–0.237]

Abbreviations: ACE, advanced cohort engine; CDW, clinical data warehouse; OR, odds ratio.

We approximated the RAM usage for a given number of patient records based on the Stanford OMOP CDM. The sum of the number of rows of all the tables containing medical events, including person, visit, condition, procedure, measurement, drug exposure, note, and note text annotation records is 2.176 billion rows. When the data from this CDM are converted to the ACE datastore, ACE requires 37GB of RAM to store and query these records. Therefore, the approximate ratio is 58 million source data rows per 1GB of RAM usage. It should be noted that this ratio may change depending on the distribution of specific kinds of records (eg, measurement records sometimes contain more data than diagnosis code records, and thus require more memory). We are able to achieve such low memory usage using techniques described in US Patent Application US20200057767A1.^23^

## DISCUSSION

A learning health system based on clinical practice, and which can tailor patient care based on past experience, requires the ability to effectively search and retrieve patient data. While many clinical data search and retrieval tools have been previously developed—some to support specific clinical applications^72^ and others to support data access for a variety of uses^21^—most do not support a conversational approach to medical data search first envisioned in the 1970s.^73^ The majority of current tools are built on top of relational databases, with form-based query interfaces which have a number of technical shortcomings and challenges for clinical search use cases highlighted in the Methods.

ACE was designed to address these challenges while excelling in performance and versatility. ACE is built on an in-memory patient object datastore to provide fast and expressive search functionality. ACE’s TQL enables the execution of complex searches with detailed temporal criteria across the common data types in electronic health records and claims. Search result validation is possible using the ACE Explorer’s graphical representation of patient timelines. ACE supports ontology-based querying by using parent-child hierarchies among disease and medication codes at search time. ACE uses OHDSI vocabularies as the backbone for this automated query expansion, and thus can be used across OHDSI sites that have OMOP CDM datasets, and with any terminologies and ontologies that are mapped to the OHDSI vocabularies. Setting up an ACE endpoint requires a software engineer, and ACE does require users to learn a new query language and understand temporal algebra. However, these requirements are comparable to that of any clinical data search tool that requires similar effort to expose clinical data. We have created extensive documentation and training material, which accompany this manuscript to support users and engineers.

ACE, and our study of its use, also has limitations. Firstly, ACE does not provide data error checking or cleaning functionality. It is assumed that any ingested data is sufficiently processed and quality-checked prior to ETL. In a similar vein, ACE searches are limited to the temporal resolution of the source data. Taking advantage of additional temporal information, which is only available in clinical notes^74^ during search, would require sites to run their own text processing tool. Secondly, our evaluation of ACE’s functionality and performance did not include a formal qualitative study to assess the information needs of intended users, and whether ACE satisfies those needs, or assessment of ACE’s efficiency in terms of information retrieval. Such studies are an important part of future work we hope to enable by widely sharing the tool. Thirdly, our evaluation of ACE’s query speed and performance is purely to illustrate the efficiency of querying. We did not include an exhaustive comparison to optimized SQL engines or cluster configurations.

The performance evaluation experiments demonstrate that ACE is highly performant even over data from hundreds of millions of patients and substantially reduces query time in comparison to a relational database query operating over the OMOP CDM. ACE combines a TQL and fast search over its patient object datastore to enable a conversation with clinical data. This conversational approach, in combination with user interfaces that provide both cohort summary and patient-level views of the data, use of knowledge graphs for result expansion, and configurable output formats support fast electronic phenotyping, cohort-building and extraction, which in turn enable data labeling, downstream analyses, clinical trial recruitment, and learning from aggregate patient data. Each of these activities contribute to the vision of a learning health system in a distinct manner. More efficient clinical trial recruitment shortens the time from identifying potentially effective therapies to assessment of their safety to their availability to patients and reduces trial costs.^75–78^ On-demand analyses of aggregate patient data via avenues such as our Informatics Consultation Service help clinicians provide the best care for their patients.^58^^,^^79^ Low latency data vending services enable data-driven operational decision-making and clinical research to advance the science and practice of medicine.^80–83^ Enabling machine learning via scalable data labeling services facilitates broad adoption of artificial intelligence to improve healthcare.^67^^,^^84–86^

## CONCLUSION

We presented a paradigm for scalable search of longitudinal patient records via an expressive TQL coupled with an in-memory patient object datastore. We implemented this capability in the form of a search tool, the Advanced Cohort Engine (ACE). ACE’s scalability and performance support a multitude of clinical and informatics use cases including cohort-building and extraction, quality metric reporting, clinical trial recruitment, labeling data for machine learning applications, and decision support based on learning from aggregate patient data. ACE ingests data from a standard schema (the OMOP CDM), to make this tool broadly accessible.

## FUNDING

This work was supported by the National Institutes of Health, grant number R01LM011369-06.

## AUTHOR CONTRIBUTIONS

AC and VP are equal contributors to this work. AC led the writing of the manuscript under the direction of NHS and contributed to the design and iterative development of ACE. VP is the architected and wrote the code of the ACE search engine, conducted experiments to evaluate ACE performance, and wrote sections of the manuscript. JDP and VP conducted experiments to compare ACE performance to a SQL database search tool. SG and JB contributed to the design and iterative development of ACE. NHS directed all research and development of ACE. All authors read and approved the manuscript.

## SUPPLEMENTARY MATERIAL

Supplementary material is available at *Journal of the American Medical Informatics Association* online.

## DATA AVAILABILITY

Data available upon request.

## CONFLICT OF INTEREST STATEMENT

None declared.

## Supplementary Material

ocab027_Supplementary_Data
