# Supplementary material for: ACE: the Advanced Cohort Engine for searching longitudinal patient records
Source: J Am Med Inform Assoc. 2021 Mar 13;28(7):1468–79. doi: 10.1093/jamia/ocab027 (PMC8279796; doi:10.1093/jamia/ocab027)
Supplement: ocab027_Supplementary_Data [file ocab027_supplementary_data.docx]

# Supplementary Information

## ACE in-memory datastore and ETL

Generating an ACE in-memory datastore of patient objects involves an extract-transform-load (ETL) process to convert patient records from a data source into the ACE datastore format. This ETL process consists of the following steps:

1. Download data from a data source (e.g. a SQL database, flat files or Apache Avro files).
2. Materialize source data dictionaries as well as join operations between different tables. Sort the data to enable construction of individual patient data objects.
3. Use hierarchical information from source data dictionaries (e.g. disease groupings from ICD) to expand source data records (e.g. expand patient records of ICD9 250.00 to include ICD9 250). Generate individual patient data objects.
4. Generate feature indices that map each patient object to the feature instances it contains, to enable fast lookup.
5. Convert patient data objects into compressed in-memory objects that do not require de-serialization.

Though ACE is not limited to any one source data schema, the preferred data schema is the OMOP CDM. Once a dataset is available in the OMOP CDM (a custom process that may itself require substantial time to design and implement an ETL process), extraction into ACE is a one-time process that is relatively quick. For example, on a dataset of 2.8 million patients, the ETL process from OMOP CDM to ACE database requires less than 6 hours. The ETL process enables ACE’s high performance search, but the data processing it involves precludes ‘live’ search of patient data as it is input by clinicians into their health record using software such as Epic’s EpicCare. Figure 1 summarizes the ETL process and possible server configurations. Code for the ETL from an OMOP CDM database to the ACE data store is included in the ACE distribution.


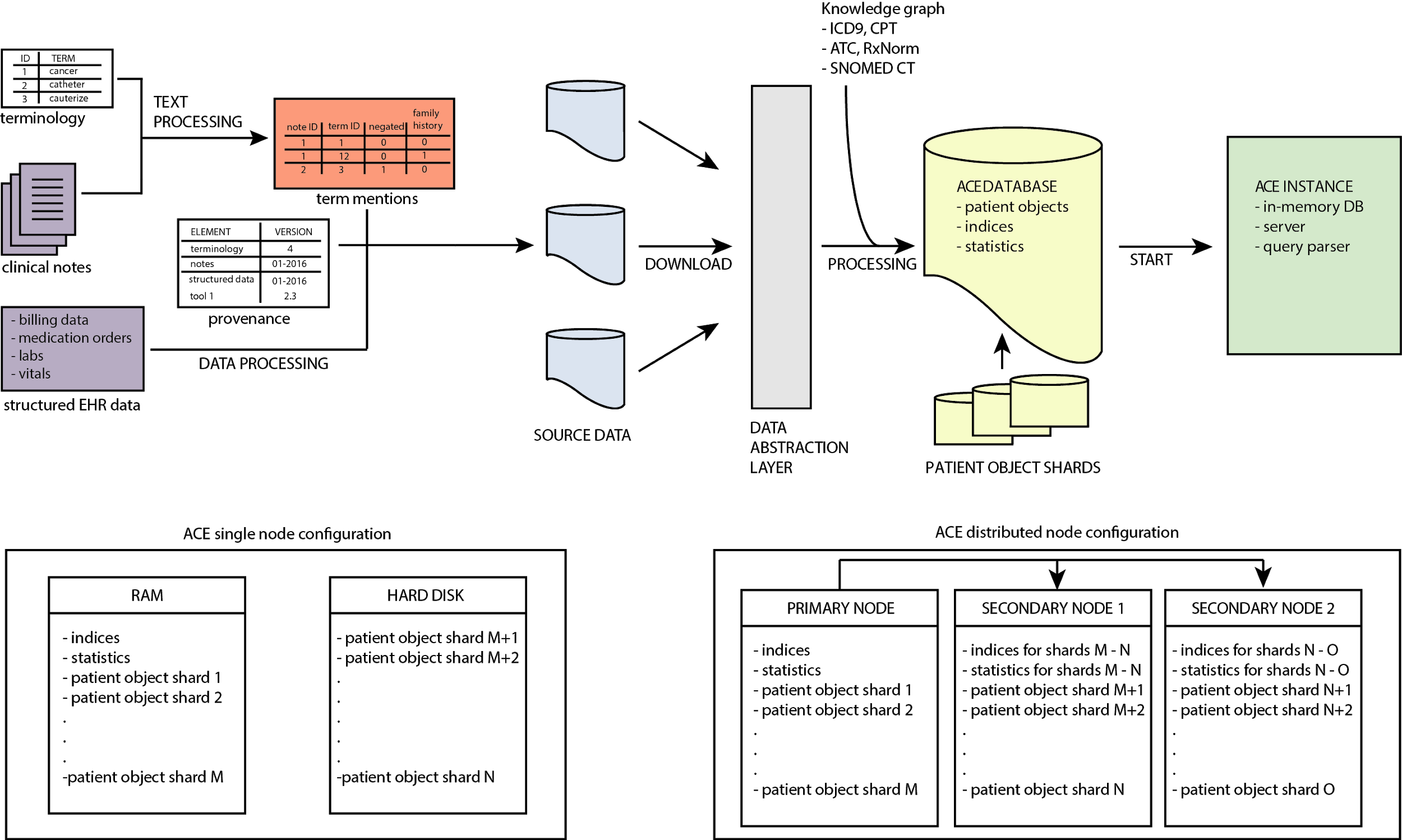


**Figure S1.** Overview of the ETL process to ingest source data and generate an ACE datastore of patient objects, and expose via an ACE server (top), with either a single node or distributed node configuration (bottom).

## TQL documentation

The Advanced Cohort Engine (ACE) uses a temporal query language (TQL) to search and extract patient data.

ACE uses a data model that structures patient records in object format where the patient object contains the entire timeline of events for the patient. Features include:

- medications records (RX, ATC, DRUG)
- measurements (LABS, VITALS, MEASUREMENT)
- CPT codes (CPT)
- ICD9 and ICD10 codes (ICD9, ICD10)
- demographics (RACE, GENDER, ETHNICITY, DEATH)
- department information (DEPARTMENT)
- enrollment (ENROLLMENT)
- visit types (VISIT TYPE)
- term mentions extracted from the full text notes (TEXT, ~TEXT, !TEXT, NOTE)
- note types (NOTE TYPE)
- snomed codes, if available (SNOMED)
- primary diagnostic codes if available (PRIMARY)
- time information (YEAR, DATE)

### Temporal Query Language

The temporal query language (TQL) allows users to search patient data by restricting values for any record type, for example to find all patients with a diagnosis of liver cancer or all patients who were prescribed the drug metformin. The query language supports boolean and temporal commands to combine queries for specific patient features into more complex queries, for example to find all female patients of Asian descent who have had a diagnosis of hypertension.

TQL Boolean commands are AND (find patients with all the specified features occurring sometime in patient’s record), OR (find patients with at least one specified feature occurring sometime in patient’s record) and NOT(find patients with no mention of the specified feature).

TQL temporal commands include INTERSECT (find patients with specified features occurring at the same time), UNION (find patients with specified features, any of which may have occurred) and SEQUENCE (find patients with specific features that occur in sequence, one after the other). Additional commands allow for searching by medical constructs such as HISTORY OF, NEVER HAD, or NO HISTORY OF.

In the ACE datastore, each patient’s record is organized on a timeline. The timeline is built of features (eg labs, vitals, hospital visits) and their associated dates. The beginning of the timeline is the first time the patient appears in the EHR and the end is his/her last appearance in the EHR. Dates are measured as the number of days since the patient’s birth. On the timeline, all data features are associated with a start date/time and an end date/time. The duration of time between the start and end is the *time interval (TI).* If the start date/time is the same as the end date/time, that is considered a *time point (TP)* ***(Figure 1)***. For example, consider an inpatient hospital admission for a patient. If the inpatient admission occurs from January 1, 2010, to January 4, 2010, then the data feature VISIT TYPE=“Inpatient” would have a TI = 4 days. If during that admission, the patient received a diagnosis of diabetes (ICD9=”250”), then the data feature ICD9=”250” would also have a TI = 4 days. If a patient had a WBC level drawn on February 1, 2011, then the data feature LAB=”WBC” would have TP = February 1, 2011. If two records of the same event type occur in overlapping time intervals, for example a record of an inpatient visit with a start date of January 12, 2010, and an end date of January 14, 2010, and a second inpatient visit with a start date of January 13 2010 and an end date of January 16, 2010, ACE will create a *computed time interval (CTI)* for an inpatient visit that spans January 12 to January 16, 2010, and this single CTI will be returned by a query for VISIT TYPE = “Inpatient”.


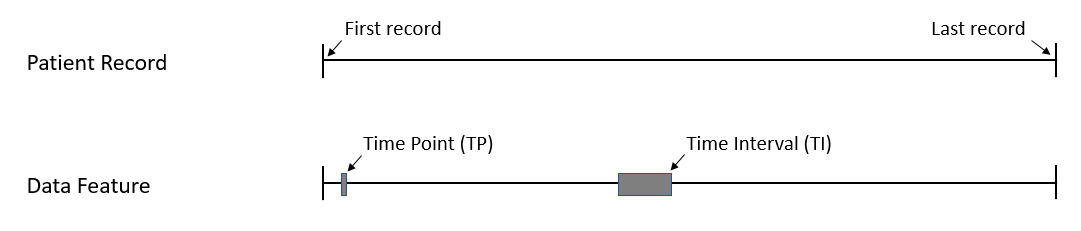


**Figure 1. Example of a patient record, with a time point (TP) and time interval (TI) for a generic data feature.**


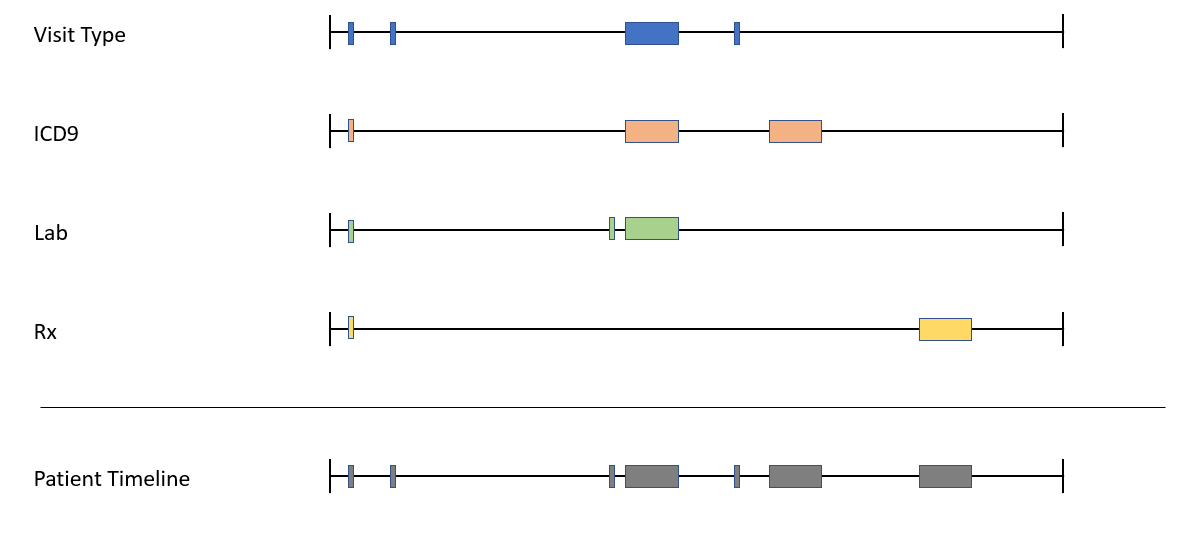


**Figure 2. Example of a patient timeline showing separate data features (e.g. Visit Type, ICD9). TIs and TPs from individual data features are combined to make up the patient timeline.**

The commands you use in a search determine the result type that is returned. Results can either be a set of booleans (true/false), which are converted to entire patient timelines, or time intervals (a range of time offsets for which a given feature is true). If a temporal command is used in a search, then the search will return patient time intervals. If only boolean commands are used in a search, boolean values are converted to entire patient timelines and returned. When temporal data is exported from ACE, it is ordered by TI or TP. This means that each row is a data feature associated with a TI or TP.

#### DEMOGRAPHICS

To start, let’s find all the women in the datastore.

| GENDER="FEMALE" |
| --- |

Start a new query and find all the Native Americans in the datastore.

| RACE="NATIVE AMERICAN" |
| --- |

Ethnicity can be queried as follows.

| ETHNICITY="HISPANIC" |
| --- |

Start a new query and find all the patients aged 40-45 years.

| AGE(40 years, 45 years) |
| --- |

The input is AGE(X,Y), with the default unit of age being *minutes* e.g. AGE(4, 10) returns all the patients 4-10 minutes old. Other units of age must be specified e.g. AGE(4 months, 10 months) or AGE(4 years, 10 years). AGE does not return all patients who ever had a record indicating a given age, but rather the TI when they were in the given age.

| DEATH |
| --- |

DEATH command returns a time point when the patient died. If death did not occur, the patient will not be part of the query result.

#### BASIC TEMPORAL OPERATIONS

Find all the diabetics, as defined by ICD9=”250”.

| ICD9=”250” |
| --- |

Find all the Native American women, aged 40-45 years, with diabetes. Query terms can be combined using AND or INTERSECT.

| AND(GENDER="FEMALE", RACE="NATIVE AMERICAN", ETHNICITY=”OTHER”, AGE(40 years, 45 years), ICD9=”250”) |
| --- |

AND is a Boolean command that returns all instances of Native American women, aged 40-45, who have had a diagnosis of diabetes (ICD9=”250”) at *any point*. The output of AND (and all other Boolean commands) is the entire timeline of patients who meet the search criteria. **(Figure 3)**

| INTERSECT(GENDER="FEMALE", RACE="NATIVE AMERICAN", AGE(40 years, 45 years), ICD9=”250”) |
| --- |

INTERSECT is a temporal command that returns all instances of Native American women, aged 40-45, who have carried a diagnosis of diabetes (ICD9=”250”) *between the ages of 40-45*. INTERSECT only returns instances where all the characteristics are met *at the same time*. The output of the INTERSECT command are TIs, not the entire patient timeline.


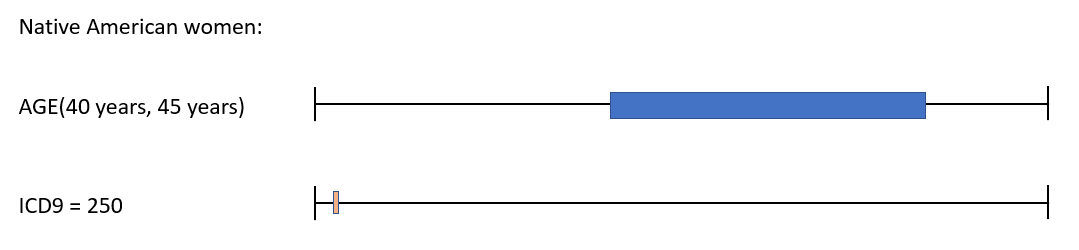


**Figure 3. Shows a Native American woman who had a diagnosis of diabetes (ICD9 = 250) before her 40th birthday and no diagnosis of diabetes during ages 40-45. Using the AND command would include this woman; using the INTERSECT command would not.**

Find all Native American women with diabetes as identified by ICD9 or ICD10 codes, who have received metformin or insulin.

| INTERSECT(GENDER="FEMALE", RACE="NATIVE AMERICAN", OR(ICD10=”E08-E13”, ICD9=”250”), OR(RX=6809, Rx=5856)) |
| --- |

The OR command is a Boolean command that looks for any of the conditions within the parentheses to be met at any time on the patient’s timeline. Note the ICD10 command that accepts ranges of input codes. The RX command accepts RxNorm identification numbers for medications. Both RX and ICD9/ICD10 commands can map typed words to appropriate codes.

You can also search prescribed drugs by class using the ATC code hierarchy, which will return any records where a drug that is a member of the specified class was prescribed. For example to find records of any lipid modifying drug prescription, the query is:

| ATC="C10A" |
| --- |

Another way to find drugs with certain criteria is the DRUG command.

| DRUG(RX=6809, ROUTE=”oral”, STATUS=”inpatient prescription”) |
| --- |

The only required parameter is the medication type (RX), which can also be specified as ATC code. Both route and status are optional, so the command can contain either or both route and status.

Now let’s find the first time patients were diagnosed with either diabetes (ICD9=”250”) or abnormal glucose tolerance test (ICD9=”648.8”):

| FIRST MENTION(UNION(ICD9=”250”, ICD9=”648.8”)) |
| --- |

This query introduces two new commands, FIRST MENTION and UNION. Both are examples of commands that return a *computed time interval (CTI),* instead of a TI or TP. This means that the time interval is computed from the input TIs and thus may be different from the input TIs (Figure 4).


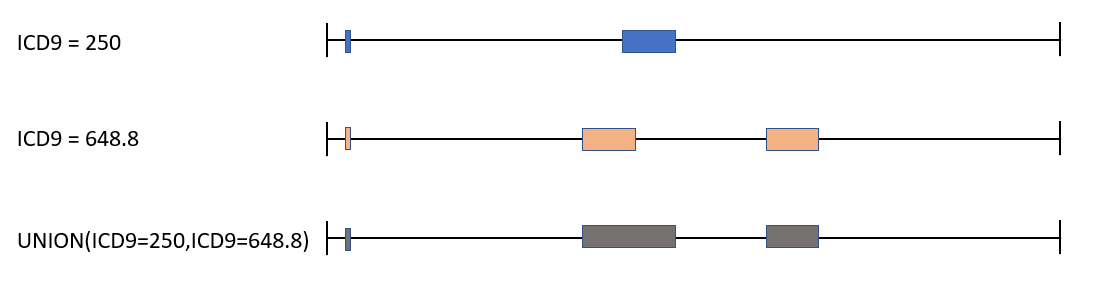


**Figure 4. Illustration of UNION command between ICD9=250 and ICD9=648.8. Resultant CTIs in gray.**

UNION combines multiple TIs. This is different from the OR command in that UNION outputs CTIs, whereas OR outputs the entire patient timeline. Notice above that the output CTIs (gray) are different from the two input TIs (blue and orange). Note that when a command returns a CTI you no longer have access to the original start or end time.


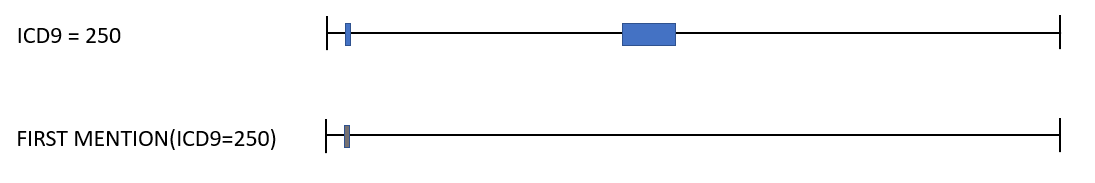


**Figure 5. Illustration of FIRST MENTION command. Resultant CTI in gray.**

FIRST MENTION outputs the TI associated with first appearance of the indicated code (ICD9 = “250” in the above example). Note that the output TI (gray) is different from the input TIs (blue), showing that FIRST MENTION returns a CTI. LAST MENTION behaves similarly.

Note that INTERSECT (seen above in Example 5) also returns a CTI.


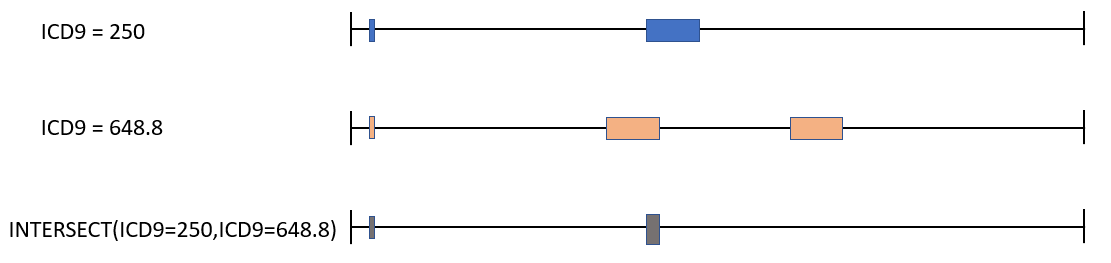


**Figure 6. Illustration of INTERSECT command between ICD9=”250” and ICD9=”648.8”. Resultant CTIs in gray.**

INTERSECT outputs a CTI only where indicated codes (in the above example, ICD9=”250” and ICD9=”648.8”) overlap.

FIRST MENTION also allows 2 arguments. FIRST MENTION (X, Y) returns a first mention of interval X within the interval Y.

Find any patient who underwent spinal fusion or had a note that mentioned ‘Scoliosis’ prior to the age of 21.

| INTERSECT(AGE(MIN, 20 years),  UNION(CPT=”22612”,CPT=”22558”,TEXT="scoliosis")) |
| --- |

The CPT (Current Procedural Terminology) command can be used to find procedures. Like ICD9/ICD10 and RX, it can map typed words to appropriate CPT codes. TEXT command searches available notes and explicitly excludes negation (i.e. excludes ‘Not concerning for scoliosis.’) and family history (i.e. excludes ‘Patient has a family history of scoliosis.’). The !TEXT command finds negated references, and the ~TEXT command finds family history references.

Find all progress notes that mention ‘diabetes’ and ‘poor compliance.’

| NOTE**(**NOTE TYPE="progress notes", AND(TEXT="diabetes", TEXT="poor compliance")) |
| --- |

The NOTE command allows you to search the text within a note type, specified by NOTE TYPE.

#### SEQUENCE

Find all of the patients who received Ceftriaxone (RX=2193) within the 5 days preceding a laparoscopic appendectomy (CPT=44970).

| SEQUENCE(RX=2193*, CPT=44970)+(-1, -5 days) |
| --- |

SEQUENCE is a sequential command that returns TIs based on their temporal relationship to other TIs. It’s a powerful command that can take multiple parameters (see full language specifications), but its basic form is SEQUENCE(X*,Y). This says return any X that precedes a Y. SEQUENCE(X,Y*) returns any Y that is preceded by an X.


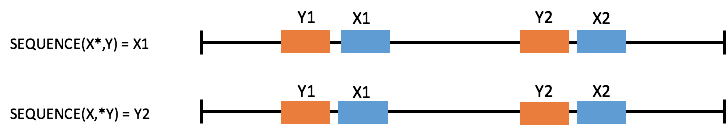


**Figure 7. Illustration of SEQUENCE command. Note that the input with the asterisk determines output.**

SEQUENCE(X*,Y)+(-A,-B) will return any X that precedes Y as long as X falls within the range defined by (-A, -B). SEQUENCE(X*,Y)-(-A,-B) will return any X that precedes Y as long as X DOES NOT fall within the range defined by (-A, -B).


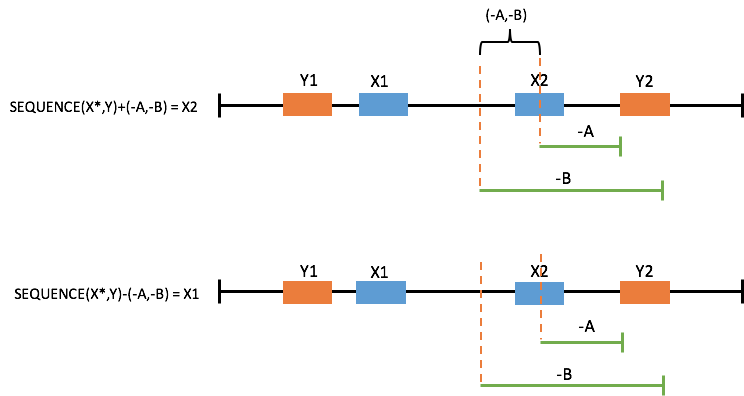


**Figure 8. Illustration of SEQUENCE command. Note that in the top example, X2 is before Y2 and falls within the range defined by (-A,-B) and is thus returned. In the bottom example, X1 is before Y2 and falls outside the range defined by (-A,-B) and is thus returned.**

#### MEASUREMENTS

ACE TQL supports both qualitative and quantitative LABS and VITALS measurement search. First, we can query for patients that had a specific LAB measurement.

| LABS(“A1C”) |
| --- |

Same command will work with VITALS measurements.

| VITALS(“BMI”) |
| --- |

The LABS and VITALS commands also accept arguments. If we are looking for a qualitative result, a single text argument is expected, for a quantitative result, a range of MIN, MAX values is expected.

| VITALS(“BMI”, 25, 30)  LABS(“A1C”, “HIGH”) |
| --- |

MEASUREMENT construct allows unified query of different measurement types.

| MEASUREMENT(VITALS(“BMI”, 25, 30))  MEASUREMENT(LABS(“A1C”, “HIGH”)) |
| --- |

#### AGGREGATE FEATURES

Aggregate features allow querying whole groups and sets of features. Main examples include ICD9, ICD10, RX, ATC, CPT, NOTES, VITALS and LABS.

| INTERSECT(UNION(ICD9, ICD10), LABS) |
| --- |

Returns all time intervals where the patient had at least one diagnostic code and a lab measurement.

ENCOUNTERS is a special calculated feature which represents a day during which a patient had a visit as specified by the visit type.

#### PATIENT LEVEL COMMANDS

Find patients that are diabetic (ICD9=”250”), but have never been diagnosed with abnormal glucose tolerance test (ICD9=”790.2”).

| VAR example_var_1 = ICD9=”250” VAR example_var_2 = ICD9=”790.2” VAR example_var_3 = DIFF($example_var_1, $example_var_2) $example_var_3 |
| --- |

For more complicated queries, variables can be defined, referenced and run. The variable is created with the VAR command. The variable is then referenced using ‘$,’ which can be used to place the variable in a separate function or to run a query with that variable.

Note the use of the DIFF(X,Y) command. This is a cohort level command that outputs a list of patient IDs that are present in X, but not in Y. The order of X and Y is important -- DIFF(Y, X) will return the patient IDs that are present in Y, but not in X. SAME(X,Y) is a similar cohort level command that outputs a list of patient IDs that are present in both X and Y. The order of X and Y is not important for SAME.

MERGE(X,Y) command simply takes the both groups of resulting patients and adds them together.

If we already know the unique patient identifiers (PID) for the patients we want to evaluate, we can use the PATIENTS command.

Take the patients with PID 3356 and PID 4423 and find out which one of them died.

| INTERSECT(DEATH, PATIENTS(3356, 4423)) |
| --- |

#### DURATIONS AND COUNTS

Another temporal command that is very useful is the DURATION command, which is used to specify that a time interval must be of a specific length. For example, to find inpatient visits of at least 5 days, the command is:

| DURATION(VISIT TYPE="inpatient", SINGLE, 5 DAYS, MAX) |
| --- |

Here, ‘SINGLE’ denotes that each single inpatient visit must be at least 5 days. Putting ‘ALL’ here instead would mean that in total, all inpatient visits for a given patient must sum to at least 5 days.

A more powerful version of this command is used to look for instances of a certain duration within other features. For example, let’s find patients who have been receiving metformin for more than 5 days while inpatient.

| DURATION(RX=6809*, VISIT TYPE="inpatient", SINGLE, 5 DAYS, MAX) |
| --- |

Notice the use of asterisk to specify which feature to return. In this case we are interested in the metformin administrations.

Similarly COUNT command allows to find instances with certain number of occurrences.

| COUNT(RX=6809, 5, MAX) |
| --- |

This example returns all the RX=6809 intervals if the patient contains more than 5 instances of these.

A more complex version of the command, allows to look for counts of instances within other features. The following version returns all the inpatient visit types that contain at least 5 RX=6809 instances. Putting ‘ALL’ keyword instead of the ‘SINGLE’ would return all the inpatient visit types if the overall count of RX=6809 for all of them *combined* is greater or equal to 5.

| COUNT(RX=6809, VISIT TYPE=”inpatient”*, SINGLE, 5, MAX) |
| --- |

#### CREATION OF TIME INTERVALS

Obtain the entire patient timeline since the first instance of receiving a diagnosis of multiple sclerosis.

| INTERVAL(ICD9=”340”, END(TIMELINE)) |
| --- |

The interval command constructs a TI using two parameters. The first parameter is the start of the TI and the second is the end of the TI. Interval parameters can be numeric, in which case it constructs an interval from the time points specified. If there are multiple instances of both parameters (multiple mentions of ICD9=”340”), the INTERVAL command will generate intervals from all the combinations where start of first interval <= end of second interval.

To guarantee a single TI result,

| INTERVAL(START(FIRST MENTION(X)), END(LAST MENTION(Y))) |
| --- |

can be used.

Additional option is to use the PAIRS modifier. INTERVAL(X, Y, PAIRS) iterates all instances of X and Y from first to last and constructs intervals for which the start of X is before the end of Y.

#### MANIPULATION OF TIME INTERVALS

The time intervals of any command can be manipulated by the EXTEND BY command.


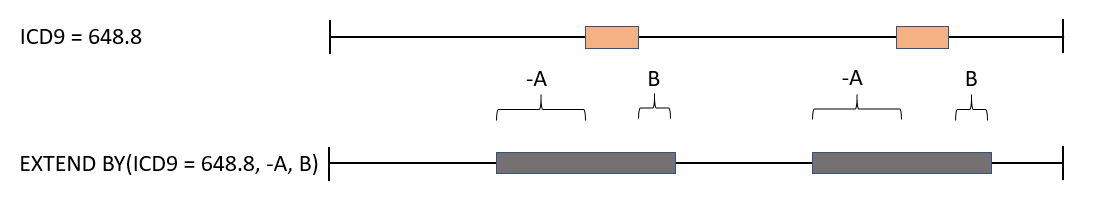


**Figure 9. Illustration of EXTEND BY command. Note that the bounds of the TIs are adjusted based on the inputs A, B.**

EXTEND BY(TI, start_time, end_time) takes an input TI and adjusts its start by the amount of time set by start_time (negative is toward the past, positive is toward the future) and adjusts its end by the amount of time set by end_time.

The arguments can further specify which part of the interval is going to be manipulated. We can move the START or the END portions of the interval.

| EXTEND BY(ICD9=”250.50”, START-1 day, START+1 day) |
| --- |

This command constructs a new time interval by taking the start of the ICD9=”250.50” and moving it 1 day into the past. The end of the interval is constructed as the start of ICD9=”250.50” interval pushed 1 day into the future. Note, that in this example the original end of the TI is ignored and the new TI is constructed solely based on the start of the original interval.

In the same way, the END keyword can be used to construct a new TI based on the end of the original TI.

Time intervals can be easily converted to time points by using the START(X) and END(X) keywords. START(RX=6809) takes all the instances of RX=6809 and returns only the start times of these instances.

#### OUTPUT OPERATIONS

After running a query in ACE, typically users want to output the data for further analysis. The most common output command is the EXPORT command. The EXPORT command outputs a tab separated file that has a defined set of columns. The values displayed in the rows are based on the input values specified by the user.

| VAR example_var_1= INTERSECT(ICD9=”250”, ICD9=”V16”) EXPORT($example_var_1, ICD9=”V16”) |
| --- |

This will download the generated file where each row is a TI associated with ICD9=”V16”. Other information contained in the file include patient ID (PID), Year, Start Age In Days (of TI), and End Age In Days (of TI). If a specific ICD9 value was not provided (e.g. EXPORT($example_var_1, ICD9)), the file would contain all TIs associated with all ICD9 values for the identified patients.

Data features that can be output in EXPORT command are: ICD9, ICD10, LABS, VITALS, CPT, RX, ATC, ENCOUNTERS, TEXT, !TEXT, ~TEXT, VISIT TYPE, NOTE TYPE, DEMOGRAPHICS**.**

All the arguments can be wrapped in COUNT(X) command which will output counts of individual events instead of events themselves.

| EXPORT(ICD9=”V16”, COUNT(CPT), COUNT(LABS)) |
| --- |

This example outputs the number of occurrences of CPT codes and number of lab measurements for each patient that had ICD9=”V16” code.

If the EXPORT command’s first argument is the DATES command, instead of age of patients, the actual dates of the TI are outputted.

| EXPORT(DATES, ICD9=”V16”, CPT) |
| --- |

This example outputs all the instances of CPT codes with dates when they occurred, instead of ages of patients when the events occurred.

Another powerful feature is the ability to restrict time of EXPORT command by specifying the time interval for which we want to perform the output.

| EXPORT(ICD9=”V16”, TIME=VISIT TYPE=”inpatient”, CPT) |
| --- |

This example takes all the patients that had ICD9=”V16” and outputs all the instances of CPT code they had during inpatient visits.

It is also possible to restrict the time interval of interest by specifying the start and end times.

| EXPORT(ICD9=”V16”, START=FIRST MENTION(ICD9=”250.50”), END=”DEATH”, LABS) |
| --- |

The output will generate a file for all the patients that had ICD9=”V16” and will output all the labs done to the patients between the first mention of diabetes ICD9 code and death.

For additional clarity, it is possible to name the parameters we want to output.

| EXPORT(ICD9=”V16”, “METFORMIN”=INTERSECT(RX=6809, VISIT TYPE=”inpatient”)) |
| --- |

The output time intervals will now be named “METFORMIN” for easier reference.

OUTPUT(X) is another output command. OUTPUT(X) produces only PIDs and the TI associated with that PID. Output columns are PID, Start Age in Days, End Age in Days.

| VAR example_var_1= INTERSECT(ICD9=250, ICD9=V16) OUTPUT($example_var_1) |
| --- |

Lastly, the EVENT FLOW(X) command exports the specified cohort into the EventFlow CoCo program format <https://hcil.umd.edu/coco/>.

#### COMPARISON

Comparison on time intervals can be done using the EQUAL(X, Y) or IDENTICAL(X, Y) commands. EQUAL returns time intervals that have the same starts and ends in both X and Y and IDENTICAL returns all TI in X only if X and Y have all the TI identical. If there is one missing or one extra in either one, IDENTICAL returns no time intervals.

To perform a comparison with an empty time interval, NULL command can be used.

| IDENTICAL(ICD9=”250.50”, NULL) |
| --- |

Returns the entire time interval for patients that did not have ICD9=”250.50”.

#### CONVENIENCE COMMANDS

Convenience commands are commands that technically do not need to exist, since they can be expressed using the already existing commands. They have been added to the language to improve the readability of the language as such.

##### HISTORY OF, NEVER HAD

HISTORY OF(X) returns at TI from the first mention of X until the end of the record. NO HISTORY OF(X) returns at TI from the beginning of the patient's chart to the first instance of X or the patient’s entire timeline if no X occurred.

Find patients who had a history of DVT of the lower extremity (ICD9=”453.4”) prior to having total knee arthroplasty (CPT=”27447”).

| INTERSECT(HISTORY OF(ICD9=”453.4”), CPT=”27447”)) |
| --- |

NEVER HAD(X) returns all patients that never had a mention of X.

##### COMPLEX INTERSECTION

In order to improve readability of the INTERSECT commands (especially the more difficult cases), the following patterns have been created:

Return all instances of ICD9=”453.4” which intersect CPT=”27447”. The interesting thing here is, that instead of INTERSECT command, here we return not just intersection of these two features, but **full** time intervals of ICD9 code that fulfil the criteria of intersecting the CPT code.

| RETURN ICD9=”453.4” INTERSECTING CPT=”27447” |
| --- |

Adding the keyword NOT allows us to return all instances of ICD9=”453.4” that **do not** intersect CPT=”27447”.

| RETURN ICD9=”453.4” **NOT** INTERSECTING CPT=”27447” |
| --- |

Let’s return all ICD9 codes that intersect the desired CPT code only if they NEVER intersect during the patient’s timeline. This means that if there is at least 1 intersection of these two codes, no interval will be returned.

| RETURN ICD9=”453.4” **NEVER** INTERSECTING CPT=”27447” |
| --- |

The opposite example can also be queried. The following command will return all specified ICD9 codes only, if all of them intersect the desired CPT code. So, if there is at least one ICD9 code that does not intersect the CPT code during the patient’s timeline, nothing will be returned.

| RETURN ICD9=”453.4” **ALWAYS** INTERSECTING CPT=”27447” |
| --- |

The RETURN command has more powerful versions too. These are invoked with the ‘ALL’ and ‘ANY’ keyword.

‘ANY’ keyword means that it is true if any of the arguments is true and ‘ALL’ is true if all of the arguments is true.

The following example returns all ICD9=”453.4” codes that intersect CPT=”27447” and also intersect RX=161. If only one of these is intersected, the result is false.

| RETURN ICD9=”453.4” INTERSECTING **ALL** (CPT=”27447”, RX=161) |
| --- |

If ALL keyword is the equivalent of AND, ANY keyword is the OR in this instance. The following example returns all ICD9=”453.4” if it intersects either CPT=”27447” or RX=161.

| RETURN ICD9=”453.4” INTERSECTING **ANY** (CPT=”27447”, RX=161) |
| --- |

The ALL and ANY keywords can be used in conjunction with the NOT, NEVER and ALWAYS keywords.

An interesting example of an intersection is the CONTAINS(X, Y) command. The command returns either X or Y (specified by asterisk convention similar to SEQUENCE) if X fully contains Y (start of X and end of X is within the bounds of Y).

##### TIMELINE

RECORD END and RECORD START are convenient ways to express the time point of the start of a patient's record and its end. They are equivalent to START(TIMELINE) and END(TIMELINE).

##### FOLLOW UP

The following command returns ICD9=”250.50” time intervals that have at least one CPT code at least 5 days before.

| FOLLOW UP OF CPT 5 DAYS BEFORE ICD9=”250.50” |
| --- |

Same follow up command can be done with the AFTER keyword.

#### QUERY LIMITS

If the dataset is large enough and for a query to complete would take an unreasonable amount of time, following queries can limit the query and return partial results. These queries must be the top level queries and cannot be nested within other queries (including EXPORT / OUTPUT).

| LIMIT(1000, ICD9=”250.50”)  LIMIT(CACHED, ICD9=”250.50”)  LIMIT(100 SECONDS, ICD9=”250.50”) |
| --- |

The first command returns only the first 1000 patients (the actual number of patients might differ slightly). The second one evaluates all the patients cached in memory and returns the ones that are evaluated as true. The third one stops evaluation after 100 seconds and returns whatever number of patients that evaluated as true.

| EVALUATE(1000, ICD9=”250.50”) |
| --- |

This command evaluates 1000 patients and returns whichever ones resulted as true. LIMIT evaluates potentially more patients to return 1000 patients.

| ESTIMATE(1000, ICD9=”250.50”) |
| --- |

Estimates cohort statistics based on 1000 evaluated patients.

#### COMMENTS

A line that starts with // or # is ignored and can contain comments about the code

| # this line is ignored  // this one too  GENDER="FEMALE" |
| --- |

#### ITERATIVE EVALUATION

For a more granular query evaluation, we can iterate through each time interval using the FOR EACH construct.

| FOR EACH(ICD9=”250.50”) AS (DIABETES) {  RETURN DIABETES AS VARIABLE_1;  }  INTERSECT(VARIABLE_1, GENDER=”MALE”) |
| --- |

The example iterates through each instance of ICD9=”250.50”, and returns it as VARIABLE_1.

The FOR EACH construct iterates through the first argument (ICD9=”250.50”) and uses it in its own context under the DIABETES name. To return the results from the FOR EACH context into the main context, RETURN DIABETES AS VARIABLE_1 is used. The VARIABLE_1 is then available in the main context.

The content between curly brackets is the FOR EACH body and can contain the following commands. Each command is separated from the previous one by a semicolon

The following commands can be used in the FOR EACH body:

X=COMMAND

Allows to assign a command to a variable

| FOR EACH(ICD9=”250.50”) AS (DIABETES) {  VARIABLE=INTERSECT(DIABETES, VISIT TYPE=”inpatient”);  RETURN VARIABLE AS VARIABLE_1;  }  INTERSECT(VARIABLE_1, GENDER=”MALE”) |
| --- |

CONTINUE

Ignores subsequent commands and evaluates the next time interval in the FOR EACH loop.

| FOR EACH(ICD9=”250.50”) AS (DIABETES) {  VARIABLE=INTERSECT(DIABETES, VISIT TYPE=”inpatient”);  IF EMPTY(VARIABLE) {  CONTINUE;  }  RETURN VARIABLE AS VARIABLE_1;  }  INTERSECT(VARIABLE_1, GENDER=”MALE”) |
| --- |

In this example, if the intersection of the DIABETES time interval and VISIT TYPE=”inpatient” is empty, the result of that command is not added to the resulting VARIABLE_1, but the next time interval is evaluated.

EXIT

Ignores subsequent commands and exits the FOR EACH loop.

| FOR EACH(ICD9=”250.50”) AS (DIABETES) {  VARIABLE=INTERSECT(DIABETES, VISIT TYPE=”inpatient”);  IF EMPTY(VARIABLE) {  EXIT;  }  RETURN VARIABLE AS VARIABLE_1;  }  INTERSECT(VARIABLE_1, GENDER=”MALE”) |
| --- |

Here, the first instance where there is no intersection between DIABETES and VISIT TYPE=”inpatient” results in returning only the results stored in the VARIABLE_1 so far.

FAIL PATIENT

A more extreme version of the EXIT command causes the evaluation of the FOR EACH loop to terminate and return a FALSE (empty interval) result.

RETURN X AS Y

Stores the result of X in the current FOR EACH iteration into the Y global variable.

CLEAR X

Deletes all the results stored into the global variable.

| FOR EACH(ICD9=”250.50”) AS (DIABETES) {  VARIABLE=INTERSECT(DIABETES, VISIT TYPE=”inpatient”);  IF EMPTY(VARIABLE) {  CLEAR VARIABLE_1;  EXIT;  }  RETURN VARIABLE AS VARIABLE_1;  }  INTERSECT(VARIABLE_1, GENDER=”MALE”) |
| --- |

In this example, we delete all the results stored into the VARIABLE_1 and exit.

IF EMPTY(X) {}

Executes the code in curly brackets if the X is an empty interval.

IF !EMPTY(X) {}

Executes the code in curly brackets if the X is not an empty interval.

IF (X) == (Y) {}

Executes the code in curly brackets if the X time intervals are identical to Y

IF (X) != (Y) {}

Executes the code in curly brackets if the X time intervals are not identical to Y

It is possible to use multiple and nested FOR EACH loops. Loops are evaluated sequentially.

### Common pitfalls

1) ACE follows an order of operations.

| UNION(FIRST MENTION(ICD9=443), FIRST MENTION(ICD9=250)) |
| --- |

is not the same as

| FIRST MENTION(UNION(ICD9=443, ICD9=250) |
| --- |

Double check the order of nested logic commands to make sure the functionality is what you expect.

2) ACE interprets the underlying data and can remove patients based on what is queried. When adding features to a query patients might be dropped from analysis from a simpler query.

| *//335 patients*  INTERSECT(YEAR(2007,2007), UNION(ICD9=572.2, ICD10=K72))  *// 336 patients*  INTERSECT(YEAR(2007,2007), UNION(ICD9=572.2)) |
| --- |

3) INVERT does not equal NOT

The NOT command is Boolean and will return the patients who do not meet the criteria. NOT(ICD9=250) = all patients who did not have a mention of diabetes in their records.

INVERT is a temporal command that will return the time interval where the condition is not true (using the patient’s timeline as a reference).

This brings up an interesting point. INVERT(ICD9=250) returns the entire timeline for any patient who did not have ICD9=250 in their timeline. For the patients who did have an ICD9=250 they will return only the time intervals where the ICD9=250 was not true.

4) Combining temporal commands with Boolean parameters should be avoided.

BEFORE(AND(ICD9=250.50, RX=161), ICD9=410*)

In the above command the AND Boolean operator will return the entire timeline of patients meeting the criteria. The BEFORE command will try to then find instances where the entire timeline came before and ICD9=410; this returns a value but *not* what is desired. ACE will give you a warning when you try to do this.

5) INTERSECT requires that two or more events happened at the exact same time. For many events in the EMR this might be too strict. It is often necessary to use the EXTEND BY function to broaden the time interval.

6) Often you care about when is the first point in a patient’s timeline that a condition is true. The FIRST MENTION command will allow you to pull out this information.

7) The AGE command defaults to minutes. Always provide a unit of time when using it.

AGE (18,24) = AGE(18 MINUTES, 24 MINUTES)

8) In general phenotyping of disease does not necessarily mean that the disease is present. For example, the presence of ICD9="250" does not mean a person is diabetic. If you can add a more stringent method to identify patients that will lead to stronger results. For example, diabetic patients have records of glycemic control, or Hemoglobin A1C measurement.

9) ICD9 and ICD10 codes are generated using hierarchical information. What that means, is that if for instance code ICD9=”250.50” is present in the data, patient will also be assigned all the hierarchical parents of this code (in this case ICD9 codes “250.5” and “250”). At query time it is possible to specify whether we want to search only the original codes or also the codes generated using the hierarchical expansion.

ORIGINAL(ICD9=”250”) will return only patients for which the code “250” was directly mentioned in the data. Omitting the ORIGINAL command will return patients for which any of the hierarchical children on ICD9=”250” were mentioned in the data.

### Frequently asked questions

1. What does it mean when the query results indicate skipped patients?

There are few reasons why certain patients are not eligible for evaluation and are skipped. The main reason is that the query contains a feature for which the patient contains data that are not correct (data points before the date of birth, data points in the future, etc.). For these data points the entire feature is marked as invalid and if the query attempts to evaluate the patient with this feature, patient is skipped from evaluation.

(Example: ICD9=250.50 contains a data point in the future. The code 250.50 is marked as invalid and when attempted to evaluate, the whole patient is skipped. Other ICD9 codes can still be evaluated for the patient).

Vitals and Labs containing invalid time events are simply removed, since their temporal persistence is low, unlike ICD9, CPT, RX codes.

The second reason a patient is skipped is when trying to evaluate a NOT or INVERT query. If the patient does not contain any events of that type, it does not mean that the specific event did not occur, it is just an example of missing data and should not be evaluated.

(Example: Patient’s records do not contain any medications. Query: NOT(RX=161) will skip the patient, since it is unclear why there are no medication records. If the patient would contain at least 1 medication record, query would succeed).

## ACE API documentation

All the API requests are POST requests if request data is required, JSON object is expected.

Example using curl:

curl -d '{"patientId":1}' -H "Content-Type: application/json" -X POST http://ace.ace.com:8080/contains_patient

### Server status

Returns server status containing any error descriptions if server cannot be reached and whether server has explorer mode enabled

**endpoint**:

/status

**request**:

not required

**response**:

status: String (error message or “OK” if server runs correctly)

workshop: boolean (true if explorer is enabled)

version: String (version of the server)

datasetVersion: String (version of the dataset loaded in the ACE instance)

ipAddress: String [optional] (displayed for responses from secondary nodes to the primary)

Example:

**request**: none

**response**:

{

"status":"OK",

"datasetVersion":"OHDSI_1",

"Version":"3.3.314",

"Workshop":true

}

### Number of queries in progress

Returns the number of queries that ACE if processing at the time of query_status request

**endpoint:**

/query_status

**request:**

none

**response:**

runningJobCount: int (number of requests ACE is currently processing)

Example

**request:** none

**response:**

{

“runningJobCount”: 1

}

### Terminate all queries in progress

Stops all requests in progress and clears the request queue. Stopped requests will not return any response.

**endpoint:**

/kill

**request:**

none

**response:**

runningJobCount: int (number of requests in progress after all requests have been stopped)

Example

**request:** none

**response:**

{

“runningJobCount”: 0

}

### Confirm presence of a patient in the datastore

Returns a boolean indication about patient’s presence in the datastore

**endpoint:**

/contains_patient

**request:**

patientId: int (patient’s identification number)

**response**:

response: boolean (true if patient exists in the datastore)

Example

**request:**

{

“patientId”: 1234

}

**response:**

{

“response”: false

}

### Clear query cache

Deletes all the cached query responses

**endpoint:**

/clear_cache

**request:**

none

**response:**

cacheSize: int (number of cached responses after the clear_cache request)

Example

**request:** none

**response:**

{

“cacheSize”: 0

}

### General population statistics

Returns basic population statistics for all the patients in the datastore

**endpoint:**

/statistics

**request:**

none

**response:**

patientCnt: int (total number of patients in the datastore)

encounterCnt: int (total number of encounters in the datastore)

gender: array of objects

label: String (gender name “MALE”, “FEMALE”, etc.)

totalPatientCnt: int (total number of patients in the datastore)

totalCohortPatientCnt: int (total number of patients in the datastore)

cohortCnt: int (not used)

generalCnt: int (total number of patients with this gender)

cohortPercentage: double (not used)

generalPercentage: double (percentage of patients with this gender in the whole dataset)

race: array of objects

label: String (race name “MALE”, “FEMALE”, etc.)

totalPatientCnt: int (total number of patients in the datastore)

totalCohortPatientCnt: int (total number of patients in the datastore)

cohortCnt: int (not used)

generalCnt: int (total number of patients with this race)

cohortPercentage: double (not used)

generalPercentage: double (percentage of patients with this race in the whole dataset)

ages: array of objects

label: String (age in years)

totalPatientCnt: int (total number of patients in the datastore)

totalCohortPatientCnt: int (total number of patients in the datastore)

cohortCnt: int (not used)

generalCnt: int (total number of patients that were this age at some point in their lives. If patient

has multiple ages recorded, he will be counted in each age. So, patient with data

in ages 1, 2 and 3 will be recorded in ages 1, 2 and 3 allowing for multiple counts

of the same patient in different ages)

cohortPercentage: double (not used)

generalPercentage: double (percentage of patients that were this age at some point in their lives in the whole dataset)

encounters: array of [key, value] number pairs

key: long (number of encounters)

value: long (number of patients with this encounter count)

durations: array of [key, value] number pairs

key: long (duration of patient’s timeline in days (patient’s timeline duration is number of days from first encounter to the

last encounter)

value: long (number of patients with this timeline duration)

deaths: array of [key, value] number pairs

key: long (number of days between birth and recorded death)

value: long (number of patients with this time of death)

censored: array of [key, value] number pairs

key: long (number of days from birth to the last encounter for all the patients without recorded

death event)

value: long (number of patients with this number of days)

Example

**request:** none

**response:**

{

"patientCnt":20000,

"encounterCnt":20000,

"gender": [

{

"label":"MALE",

"totalPatientCnt":20000,

"totalCohortPatientCnt":20000,

"cohortCnt":0,

"generalCnt":10000,

"cohortPercentage":0,

"generalPercentage":0.5

},

{

"label":"FEMALE",

"totalPatientCnt":20000,

"totalCohortPatientCnt":20000,

"cohortCnt":0,

"generalCnt":10000,

"cohortPercentage":0,

"generalPercentage":0.5

}

],

"race": [

{

"label":"WHITE",

"totalPatientCnt":20000,

"totalCohortPatientCnt":20000,

"cohortCnt":0,

"generalCnt":10000,

"cohortPercentage":0,

"generalPercentage":0.5

},

{

"label":"ASIAN",

"totalPatientCnt":2309652,

"totalCohortPatientCnt":2309652,

"cohortCnt":0,

"generalCnt":4439,

"cohortPercentage":0,

"generalPercentage":0.0019219345598384518

}

],

"ages": [

{

"label":"0",

"totalPatientCnt":20000,

"totalCohortPatientCnt":20000,

"cohortCnt":0,

"generalCnt":2000,

"cohortPercentage":0,

"generalPercentage":0.1

},

{

"label":"1",

"totalPatientCnt":20000,

"totalCohortPatientCnt":20000,

"cohortCnt":0,

"generalCnt":18000,

"cohortPercentage":0,

"generalPercentage":0.9

}

],

"encounters": [

[0,1000],

[1,1000],

[2,18000]

],

"durations": [

[0,2000],

[1,2000],

[2,16000]

],

"deaths": [

[20,2000],

[21,2000],

],

"censored": [

[0,2000],

[10,2000],

[25,12000]

]

}

### Refresh list of secondary nodes, connect them to primary

Each primary server node contains a list of shards it queries for sharded data and collates the results from all the secondary nodes + primary to return a single response (distributed architecture). This list of secondary nodes is located in the primary server’s root folder called secondary.dat.

Each secondary has a line in the file:

SECONDARY;http://abc:8081

SECONDARY;http://def:80802

After modifying the secondary.dat file, to refresh the list of secondary nodes, refresh_secondary_list needs to be called

**endpoint:**

/refresh_secondary_list

**request**:

None

**response**:

error: String (errors while contacting the primary and retrieving the list of shards. If errors were not encountered, error field is omitted in the response)

count: int (number of shards attached)

secondary: array of objects

status: String (status of the secondary node, “OK” if node is running correctly)

workshop: boolean (always false for secondary nodes)

version: String (version of the secondary node)

Example

**request:** none

**response:**

{

“count”: 2,

“urls”: [

{

“status”: “OK”,

“workshop”: false,

“version”: “1.2”,

“ipAddress”: “1.2.3.4:8080”,

“datesetVersion”: “version”

},

{

“status”: “OK”,

“workshop”: false,

“version”: “1.2”

“ipAddress”: “1.2.3.4:8080”,

“datesetVersion”: “version”

}

]

}

### Autosuggest search

Performs an autosuggest search and returns top 10 suggestions (by frequency) and a number of other suggestions that are not returned. Allows performing a search within a subset of patient ids for the purposes of explorer.

**endpoint:**

/autosuggest

**request:**

query: String (search string)

position: int (position of the cursor in the string)

patientIds: array of int (patient identifiers for which to search and within which to return patient counts)

**response**:

response: array of String (autosuggest lines to populate the autosuggest search)

remaining: int (total number of responses that are not returned in the response array)

Example

request:

{

“query”: “ICD9=diab”,

“position”: 9,

“patientIds”: [1, 2, 3, 4]

}

response:

{

“response”:

[

“250.50 diabetes mellitus [2]”,

“250.55 diabetes mellitus type 2 [1]”

],

“remaining”: 1

}

### Autosuggest replace text with selected item

Takes a query text and replaces the text for which the autosuggest search returned a response with the suggested text

**endpoint:**

/autosuggest_replace

**request:**

selectedCode: String (exact string from the autosuggest_search list selected for replacement)

text: String (current text before replacement)

cursor: int (cursor position)

response: text: String (replaced text)

cursor: int (new cursor position after replacement)

Example

**request:**

{

“text”: “ICD9=diab”,

“selectedCode”: “250.50 diabetes mellitus [2]”,

“cursor”: 9

}

**response:**

{

“text”: “ICD9=250.50”, “cursor”: 11

}

### Names of codes

Returns the names of different codes (ICD9, CPT, etc.)

**endpoint:**

/dictionary

**request:**

cpt: array of String ()

icd9: array of String ()

icd10: array of String ()

atc: array of String ()

labs: array of String ()

rxNorm: array of String ()

**response:**

cpt: array of [code, text] [String, String] pairs

icd9: array of [code, text] [String, String] pairs

atc: array of [code, text] [String, String] pairs

labs: array of [code, text] [String, String] pairs

rxNorm: array of [code, text] [String, String] pairs

Example

**request:**

{

“cpt”:

[

“25000”,

“30000”

],

“icd9”:

[

“250.50”

]

}

**response:**

{

“cpt”:

[

[“25000”, “procedure code name 1”],

[“30000”, “procedure code name 2”]

],

“icd9”:

[

[“250.50”, “Diabetes mellitus type 2”]

]

}

### Perform a search query

**endpoint:**

/query

**request:**

query: String (query string to perform search on)

returnPids: boolean (return patient identifiers that satisfy the query)

returnTimeIntervals: boolean (in addition to patient identifiers, return also time when the query was true)

returnYears: boolean (in addition to patient identifiers and/or time intervals return years for start time).

Only applicable with returnTimeIntervals

returnSurvivalData: boolean (return data to calculate survival charts)

pidCntLimit: int (maximum number of patients to return)

statisticsLimit: int (maximum number of patients for which to calculate statistics (age distribution, codes, etc.)

encountersBuckets: array of int (description of encounter ranges for which to calculate encounter distribution)

durationBuckets: array of int (description of duration ranges for which to calculate duration distribution)

response:

patientIds: array of double arrays (array of triplets of doubles (patient id, start time, end time) if returnTimeIntervals is set to true, or array of arrays of doubles (patient id))

originalUnparsedQuery: String (original query string)

timeTook: long (milliseconds it took to run the query)

parsedQuery: String (normalized and parsed query)

exportLocation: String (url to a file, if response contains a link to a file)

cohortPatientCnt: int (number of patients for which the query was true)

generalPatientCnt: int (total number of patients in the datastore)

processedPatients: int (number of patients inspected to satisfy this query)

deaths: array of [key, value] number pairs

key: long (number of days between birth and recorded death)

value: long (number of patients with this time of death)

censored: array of [key, value] number pairs

key: long (number of days between birth and end of record if no death was recorded)

value: long (number of patients with this duration)

genderHistogram: array of objects

label: String (gender name “MALE”, “FEMALE”, etc.)

totalPatientCnt: int (total number of patients in the datastore)

totalCohortPatientCnt: int (total number of patients satisfying this query)

cohortCnt: int (number of patients with this gender within this query)

generalCnt: int (total number of patients with this gender in the datastore)

cohortPercentage: double (percentage of patients with this gender in this query)

generalPercentage: double (percentage of patients with this gender in the whole dataset)

raceHistogram: array of objects

label: String (race name “ASIAN”, “WHITE”, etc.)

totalPatientCnt: int (total number of patients in the datastore)

totalCohortPatientCnt: int (total number of patients satisfying this query)

cohortCnt: int (number of patients with this race within this query)

generalCnt: int (total number of patients with this gender in the datastore)

cohortPercentage: double (percentage of patients with this race in this query)

generalPercentage: double (percentage of patients with this race in the whole dataset)

icd9Statistics: array of objects

label: String (name of ICD9 code)

code: String (ICD9 code)

totalPatientCnt: int (total number of patients in the datastore)

cohortCnt: int (number of patients in this query with this ICD9 code)

generalCnt: int (number of patients in the datastore with this ICD9 code)

cohortPercentage: double (percentage of patients in this cohort with this ICD9 code)

generalPercentage: double (percentage of patients with this ICD9 code in the datastore)

cptStatistics: array of objects

label: String (name of CPT code)

code: String (CPT code)

totalPatientCnt: int (total number of patients in the datastore)

cohortCnt: int (number of patients in this query with this CPT code)

generalCnt: int (number of patients in the datastore with this CPT code)

cohortPercentage: double (percentage of patients in this cohort with this CPT code)

generalPercentage: double (percentage of patients with this CPT code in the datastore)

rxStatistics: array of objects

label: String (name of RxNorm code)

code: String (RxNorm code)

totalPatientCnt: int (total number of patients in the datastore)

cohortCnt: int (number of patients in this query with this RxNorm code)

generalCnt: int (number of patients in the datastore with this RxNorm code)

cohortPercentage: double (percentage of patients in this cohort with this RxNorm code)

generalPercentage: double (percentage of patients with this RxNorm code in the datastore)

labsStatistics: array of objects

label: String (name of LAB code)

code: String (LAB code)

totalPatientCnt: int (total number of patients in the datastore)

cohortCnt: int (number of patients in this query with this LAB code)

generalCnt: int (number of patients in the datastore with this LAB code)

cohortPercentage: double (percentage of patients in this cohort with this LAB code)

generalPercentage: double (percentage of patients with this LAB code in the datastore)

encounters: array of int (count of patients for each range specified in the encountersBuckets)

durations: array of int (count of patients for each range specified in the durationBuckets)

errorMessage: String (error message if there was an error during query execution)

warningMessage: String (warning message if warning was issued)

ages: array of objects

label: String (age)

code: String (age)

totalPatientCnt: int (total number of patients in the datastore)

cohortCnt: int (number of patients in this query with this age)

generalCnt: int (number of patients in the datastore with this age)

cohortPercentage: double (percentage of patients in this cohort with this age)

generalPercentage: double (percentage of patients with this age in the datastore)

patientStatistics: array of objects for “DRUGS”, “LABS”, “ICD”, “CPT”, “NOTES” to generate the diagram of

percentages of patients having the information about DRUGS, ICD, CPT, etc.

label: String (name of the code in a section)

code: String (code in a section)

totalPatientCnt: int (total number of patients in the datastore)

cohortCnt: int (number of patients in this query with this code)

generalCnt: int (number of patients in the datastore with this code)

cohortPercentage: double (percentage of patients in this cohort with this code)

generalPercentage: double (percentage of patients with this code in the datastore)

consoleOutput: array of String (output from a CONSOLE command)

Example

**request:**

{

“query”: “ICD9=250.50”,

“returnPids”: true,

“returnTimeIntervals”: true,

“returnSurvivalData”: false,

“pidCntLimit”: 20,

“statisticsLimit”: 20,

“encountersBuckets”: [0, 5, 10, 15, 20],

“durationBuckets”: [0, 5, 10, 15, 20],

“binary”: false

}

**response:**

{

"patientIds":

[

[333.0,30337.0,30337.0],

[957.0,36370.0,36370.0],

[957.0,36393.0,36393.0]

],

"originalUnparsedQuery":"ICD9=350.50",

"timeTook":35,

"parsedQuery":"OUTPUT(ICD9=350.50)",

"exportLocation":null,

"cohortPatientCnt":3635,

"generalPatientCnt":3309653,

"skippedPatientCnt":56,

"processedPatients":3673,

"Deaths":null,

"Censored":null,

"genderHistogram":

[

{

"label":"FEMALE",

"totalPatientCnt":3309653,

"totalCohortPatientCnt":3635,

"cohortCnt":9,

"generalCnt":3356350,

"cohortPercentage":0.0033896365560365973,

"generalPercentage":0.533869833333958

},

{

"label":"MALE",

"totalPatientCnt":3309653,

"totalCohortPatientCnt":3635,

"cohortCnt":33,

"generalCnt":3050353,

"cohortPercentage":0.0033395030736887966,

"generalPercentage":0.3536797536307333

}

],

"raceHistogram":

[

{

"label":"UNKNOWN",

"totalPatientCnt":3309653,

"totalCohortPatientCnt":3635,

"cohortCnt":3,

"generalCnt":993363,

"cohortPercentage":5.533503357833663E-3,

"generalPercentage":0.33003930333737693

},

{

"label":"WHITE",

"totalPatientCnt":3309653,

"totalCohortPatientCnt":3635,

"cohortCnt":7,

"generalCnt":837383,

"cohortPercentage":0.0039363763303353335,

"generalPercentage":0.3538559055639933

}

],

"icd9Statistics":

[

{

"label":"Hangnail with lymphangitis of finger",

"code":"L03.03",

"cohortCnt":3,

"generalCnt":3,

"totalPatientCnt":3309653,

"cohortPercentage":3.7663537389073305E-3,

"generalPercentage":0,

"residual":35.337063983636355,

"labelValue":0

},

{

"label":"Acute lymphangitis of left finger",

"code":"L03.033",

"cohortCnt":3,

"generalCnt":3,

"totalPatientCnt":3309653,

"cohortPercentage":3.7663537389073305E-3,

"generalPercentage":0,

"residual":35.337063983636355,

"labelValue":0

}

],

"encounters": [3, 3, 3, 3, 33],

"durations": [3,3,3,3,37],

"errorMessage":null,

"warningMessage":null,

"ages":

[

{

"label":"0",

"code":"0",

"cohortCnt":0,

"generalCnt":337335,

"totalPatientCnt":3309653,

"cohortPercentage":0,

"generalPercentage":0.09833733537670835,

"residual":0,

"labelValue":0

},

{

"label":"3",

"code":"3",

"cohortCnt":0,

"generalCnt":38733,

"totalPatientCnt":3309653,

"cohortPercentage":0,

"generalPercentage":0.033090390330373366,

"residual":0,

"labelValue":0

}

],

"patientStatistics":

[

{

"label":"DRUGS",

"code":"",

"cohortCnt":30,

"generalCnt":965993,

"totalPatientCnt":3309653,

"cohortPercentage":0.005533503357833663,

"generalPercentage":0.3383333386993885,

"residual":-38.369393663333896,

"labelValue":0

},

{

"label":"ICD9",

"code":"",

"cohortCnt":33,

"generalCnt":3335333,

"totalPatientCnt":3309653,

"cohortPercentage":0.005809338630705393,

"generalPercentage":0.63703835377333,

"residual":-36.783337965306556,

"labelValue":0

},

{

"label":"CPT",

"code":"",

"cohortCnt":33,

"generalCnt":3333068,

"totalPatientCnt":3309653,

"cohortPercentage":0.005809338630705393,

"generalPercentage":0.5680803863360933,

"residual":-33.85337603333385,

"labelValue":0

},

{

"label":"LABS",

"code":"",

"cohortCnt":38,

"generalCnt":836300,

"totalPatientCnt":3309653,

"cohortPercentage":0.003979353333033395,

"generalPercentage":0.3663633336893033,

"residual":-35.903738363936353,

"labelValue":0

},

{

"label":"NOTES",

"code":"",

"cohortCnt":33,

"generalCnt":3690033,

"totalPatientCnt":3309653,

"cohortPercentage":0.005809338630705393,

"generalPercentage":0.7337367363563383,

"residual":-53.03376633937758,

"labelValue":0

}

],

"consoleOutput":[]

}

### Query a list of patient ids

For a specific query, returns only the list of patient ids without any other information. This response is NOT a json response, just a flat \n delimited list of patient ids.

**endpoint:**

/pids

**request:**

query: String (query string)

pidCntLimit: int (how many patients to return)

returnPids: boolean (must be set to true)

binary: boolean (response is either a set of binary integers or a textual response delimited by \n character)

**response:**

text string of list of patient ids delimited by ‘\n’ character

Example

**request:**

{

“query”: “ICD9=250.50”,

“pidCntLimit”: 20,

“returnPids”: true,

“binary”: false

}

**response:**

1\n2\n3\n4\n

### Dump entire patient record

**endpoint:**

/dump

**request:**

patientId: int (patient identifier to dump)

patientIds: array of int (list of patient identifiers to dump)

icd9: boolean (return ICD9 data in the dump)

icd10: boolean (return ICD10 data in the dump)

departments: boolean (return DEPARTMENT data in the dump)

cpt: boolean (return CPT data in the dump)

rx: boolean (return RxNorm data in the dump)

snomed: boolean (return SNOMED data in the dump)

notes: boolean (return NOTES data in the dump)

visitTypes: boolean (return VISIT TYPES data in the dump)

noteTypes: boolean (return NOTE TYPES data in the dump)

encounterDays: boolean (return ENCOUNTER DAYS data in the dump)

ageRanges: boolean (return AGE data in the dump)

labs: boolean (return LABS data in the dump)

vitals: boolean (return VITALS data in the dump)

atc: boolean (return ATC to RxNorm mapping data in the dump)

selectionQuery: String (evaluate this query for each patient and return only data within this time interval)

containsStart: boolean (for selectionQuery return only intervals that begin during the selectionQuery or any combination of containsStart / containsEnd)

containsEnd: boolean (for selectionQuery return only intervals that end during selectionQuery)

**response:** array of objects

patientId: int (patient identifier)

recordStart: int (time of first recorded event in minutes since date of birth)

recordEnd: int (time of last recorded event in minutes since date of birth)

death: int (time of death in minutes since date of birth. Zero if no date of birth)

gender: String (gender label)

race: String (race label)

ethnicity: String (ethnicity label)

icd9: array of [key String, value String array] pairs

key: String (ICD9 code)

value: start date in minutes since DOB, end date in minutes since DOB, PRIMARY diagnosis / OTHER

cpt: array of [key String, value int array] pairs

key: String (CPT code)

value: start date in minutes since DOB, end date in minutes since DOB

rx: array of [key String, value of String array]

key: String (RxNorm code)

value: start date in minutes since DOB, end date in minutes since DOB, route, status

snomed: array of [key String, value String array] pairs

key: String (SNOMED code)

value:start date in minutes since DOB, end date in minutes since DOB, PRIMARY diagnosis / OTHER

negatedTerms: array of [key String, value String array] pairs

key: String (negated term mention)

value: start date in minutes since DOB, end date in minutes since DOB, note type

positiveTerms: array of [key String, value String array] pairs

key: String (positive term mention)

value: start date in minutes since DOB, end date in minutes since DOB, note type

fhTerms: array of [key String, value String array] pairs

key: String (family history term mention)

value: start date in minutes since DOB, end date in minutes since DOB, note type

visitTypes: array of [key String, value int array] pairs

key: String (visit type)

value: start date in minutes since DOB, end date in minutes since DOB

noteTypes: array of [key String, value int array] pairs

key: String (note type)

value: date in minutes since DOB

atc: array of [key String, value int array] pairs

key: String (atc class code)

value: RxNorm codes belonging to this class

labs: array of [key String, value String array] pairs

key: String (lab code)

value: date in minutes since DOB, interpreted lab value (UNDEFINED, HIGH, LOW, etc.)

labsRaw: array of [key String, value String array]

key: String (lab code)

value: date in minutes since DOB, numerical value of the lab

vitals: array of [key String, value String array]

key: String (name of vitals measurement)

value: date in minutes since DOB, numerical value of the measurement

yearRanges: array of [key String, value int array] pairs

key: String (year)

value: start date in minutes since DOB in which patient had date in this year, end date in minutes since DOB in which patient had date in this year

encounterDays: array of int (list of start dates and end dates in minutes since DOB of

the days during which the patient had at least one encounter)

ageRanges: array of int ( list of start dates and end dates in minutes since DOB during

which the patient had at least one event)

error: String (error string)

Example

**request:**

{

"patientId":5538,

"icd9":true,

"icd10":false,

"departments":false,

"cpt":true,

"rx":true,

"snomed":true,

"notes":true,

"visitTypes":true,

"noteTypes":true,

"encounterDays":true,

"ageRanges":true,

"labs":true,

"vitals":true,

"atc":true,

"containsStart":false,

"containsEnd":false,

"Compress":false

}

response:

{

"binaryResponse":false,

"patientId":2,

"recordStart":43525440,

"recordEnd":44115840,

"death":0,

"gender":"FEMALE",

"race":"ASIAN",

"ethnicity":"NON-HISPANIC",

"icd9":

{

"440-449.99":["112","112","OTHER"],

"447.4":["223","224","OTHER"]

},

"cpt":

{

"99284":[1234,1234,223,225],

"99283":[223, 223, 255, 256]

},

"rx":

{

"10689":["123","125","oral","inpatient administered",

"221","222","oral","outpatient"],

"1089":["223","226","oral","inpatient administered",

"228","229","oral","outpatient"]

},

“negatedTerms":

{

"edema":["25440","97328","ed provider notes",

"85920","55843","ed provider notes",

"88800","04533","ed provider notes"],

"sleep disturbance":["25440","97328","ed provider notes"]

},

"visitTypes":

{

"hospital encounter":[5440,5440,5920,5920,7360,7360]

},

"noteTypes":

{

"ed provider notes":[440,920,800],

"hip 1 view left and pelvis":[440,920],

},

"atc":

{

"s01h":[6387, 2213],

"A06aa02":[82003]

},

"labs":

{

"30180-4":["43525440","UNDEFINED","43585920", “HIGH”]

},

"labsRaw":

{

"30180-4":["43525440","0.6","43585920","0.6","43588800","0.5"]

},

"vitals":

{

"Heart Rate (Pulse)":["43587360","69.0","43585920","73.0","43525440",

"80.0"]

},

"yearRanges":

{

"2016":[43525440,44115840]

},

"encounterDays": [112, 223, 228, 300],

"ageRanges":[112, 333]

}

### Custom patient data dump

For a list of pids, generate a custom data dump

**endpoint:**

/custom_dump

**request:**

patientIds: array of long (ids of patients to retrieve data for)

header: array of String (file header. Number of columns must match columns array)

columns: array of String (data to generate. See data dictionary)

columnSeparator: String (optional. Default value is \t)

rowSeparator: String (optional. Default value is \n)

quote: String (optional. Quote for string values)

**response:**

String data generated by the request

Data dictionary:

General columns:

PID patient_id

YEAR year event took place

START start time of event (in minutes)

END end time of event (in minutes)

RECORD_START record start (in minutes)

RECORD_END record end (in minutes)

DEATH death time (in minutes)

Specific columns:

GENDER

RACE

ETHNICITY

ICD9 ICD9 code

ICD10 ICD10 code

Modifiers:

PRIMARY 1 if the code is primary, 0 if not

ORIGINAL 1 if the code is original (not generated by hierarchy), 0 if generated by hierarchy

CPT CPT code

RX RxCui code

Modifiers:

STATUS medication status

ROUTE medication route

TEXT positive text mention

NEGATED_TEXT negated text mention

FAMILY_TEXT family text mention

Modifiers:

NOTE_TYPE note type name

VISIT_TYPE visit type name

LABS labs name

Modifiers:

VALUE lab value

VITALS vitals measurement

Modifiers:

VALUE vitals value

Only one specific column can be used in a query and only modifiers coupled with the specific column can be used. This means that a column definition cannot contain, for example, CPT and ICD at the same time and CPT cannot be used with the STATUS

or ROUTE.

Example

**request:**

{

“patient_ids”: [1, 2, 3],

“header”: [“pid”, “start”, “end”, “year”, “rxCui”, “route”, status”],

“columns”: [“PID”, “START”, “END”, “YEAR”, “RX”, “ROUTE”, “STATUS”]

}

**response:**

pid\tstart\tend\tyear\trxCui\troute\tstatus\n1\t300435\t300438\t2014\t161\toral\tinpatient\n

**Error response:** ERROR: Error message

## Comparison of ACE with existing search engines

In developing ACE, we held meetings with medical domain experts to ascertain their search needs. In addition, we conducted a literature review of medical search engines and query tools to summarize currently available systems and system designs. The features we used to compare ACE with existing systems are based on use cases identified in our meetings with domain experts and those described in the literature. The features summarized in Table S1 are the union of these approaches. Most existing tools use SQL or NoSQL database engines and do not support, or have a limited support for, temporal queries. Most do not provide many options to output results into formats directly usable in the most popular tools.

**Table S1.** Medical search engines and the features they support.

| **Feature category** | **Feature** | **ACE** | **Data**  **Sphere** | **X**  **-Search** | **EMERSE** | **UPDBL** | **Dr.**  **Warehouse** | **EHDViz** | **DEDUCE** | **Leaf** |
| --- | --- | --- | --- | --- | --- | --- | --- | --- | --- | --- |
| Domain specific feature support | Diagnosis | X | X | limited |  | X | X | X | X | X |
|  | Procedure | X | X |  |  | X | X | X | X | X |
|  | Medication | X | X | X |  | X | X | X | X | X |
|  | Measurement | X | X | X |  |  | X | X | X | X |
|  | Processed note | X |  |  |  |  |  |  |  |  |
|  | Full text note |  |  |  | X |  | X |  |  |  |
|  | Demographics | X | X | X |  | X | X | X | X | X |
|  | Visit detail | X | X |  |  |  |  |  | X |  |
| Domain agnostic feature support | Ontology hierarchy | X | X |  | X |  | X |  |  | X |
|  | Temporal querying | X |  |  |  |  | limited |  |  | X |
| Search output | Produces cohort counts | X | X | X | X | X | X |  |  | X |
|  | Produces patient IDs | X |  | X | X |  |  |  | X | X |
|  | Allows data export | X |  |  |  |  |  |  | X |  |
|  | Data visualization | X |  | X | X |  | X | X |  | X |
|  | REST API | X |  |  |  |  |  |  | X |  |

The search engines summarized alongside ACE are listed below with a citation to the relevant publication and a short description.

- **EMERSE** [[6]](https://paperpile.com/c/63NfSP/SUty): A case sensitive full text search engine built on Lucene with added synonym support.
- **UPDB** [[9]](https://paperpile.com/c/63NfSP/kCpq): Utah Population Database search engine. Data is aggregated from various registration records and pooled with medical data.
- **DEDUCE** [[16]](https://paperpile.com/c/63NfSP/b5Gs): A search engine with full text search support allowing for simple UI driven query generation with no temporal support.
- **EHDViz** [[17]](https://paperpile.com/c/63NfSP/XAXf): A set of open-source tools allowing to visualize quantitative features of patients (labs, vitals) for specific patients.
- **DataSphere** [[10]](https://paperpile.com/c/63NfSP/Mthl): A NoSQL MongoDB database search engine with ontology support, but high resource consumption.
- **X-Search** [[11]](https://paperpile.com/c/63NfSP/FpOK): A search engine that allows search on multiple different harmonized and normalized datasets. UI generated queries are translated into SQL and executed on multiple SQL databases.
- **Dr Warehouse** [[18]](https://paperpile.com/c/63NfSP/DBxj): A temporal search engine on text notes and codes primarily useful for cohort exploration.
- **Leaf** [[13]](https://paperpile.com/c/63NfSP/BXSR): A form-based temporal search engine designed to estimate patient cohort sizes prior to a study. Allows querying arbitrary database schemas with ontology support.

In addition, we describe search engines and related tools listed below which, though relevant as tools to support clinical data search and/or retrieval, were not compared with ACE because their functionality or features are quite different. We include them here for completeness and to point readers to related tools.

- **Visual Query Tool** [[8]](https://paperpile.com/c/63NfSP/r4eW): A search engine that queries a MS.SQL database via Java / MS.COM middle tier with HTML (ActiveX) front end. The queries can be executed via text interface (undocumented) or HTML interface. Queries consist of a hierarchical tree of concepts (diagnostic, procedure codes, demographics, etc.) and they can be intersected with other concepts. Each concept selection can be delimited by dates. The interface is a simple representation of a Venn diagram. Its main limitation is low granularity in temporal queries. Only intersections are supported and no meta constraints are allowed (first instance, etc.). A useful feature is that patients are indexed from multiple facilities (note that this is not a feature of the search engine, but rather a feature of the underlying data warehouse).
- **The EHR Search Engine** [[15]](https://paperpile.com/c/63NfSP/2wNS): A search engine where a free text query is parsed and mapped to concepts in UMLS and suggestions for more accurate search terms are provided. Too general concepts (for example ‘patient’) are filtered out and relevant concepts are expanded to include other variants of the terms synonymous with the search terms. These are ordered by relevancy and presented to the user. Only a subset of UMLS semantic groups is used to maximize the usefulness of presented terms.
- **Federated Query Tool** [[15]](https://paperpile.com/c/63NfSP/2wNS): Enables aggregation of queries across multiple data warehouses. Each warehouse contains deidentified i2b2 data. Each query is federated across all of the datasets. The main disadvantage is that only numbers of patients are returned and these are further obfuscated by adding small random numbers. No additional results are returned.
- **DISCERN** [[19]](https://paperpile.com/c/63NfSP/bcdm): DISCERN ingests HL7 messages, converts them into XML messages. These messages are then processed and in combination with other queries (such as queries to database) it is determined if the patient who was the subject of the HL7 message is eligible for a study. If that is true, the result is stored into a database and a message to DISCERN users is sent out. The advantages of this approach are that it operates over real time data with enough granularity to determine eligibility criteria which would be difficult to do in a data warehouse (time sensitive data or metadata are not normally captured in a data warehouse).
- **ECLECTIC (query language)** [[20]](https://paperpile.com/c/63NfSP/Xl5G): ECLECTIC is a high level language that allows expressing eligibility criteria in a human readable schema agnostic way. All events are considered to have zero length and therefore it provides only basic temporal expressions: BEFORE, AFTER, NOW, FIRST, LAST, EQUALS and binary operators AND, OR, NOT.

## Query writing times using TQL and BigQuery SQL on OMOP CDM

To compare ACE TQL with BigQuery SQL, we asked a research engineer with expertise using BigQuery and the OMOP CDM to write and execute the SQL queries needed to express the type II diabetes cohort definition in Box 1. Table S2 summarizes the results of executing this cohort definition using ACE TQL in comparison to SQL over a BigQuery instance formatted using the OMOP CDM v5.3. Boxes S1-S4 contain the corresponding SQL queries necessary to execute this cohort definition over the same data available via a Google Bigquery instance, with the data structured using the OMOP CDM v5.3.

Cohort definition: *Male patients over 65 years old who are type II diabetic (defined by at least 2 occurrences of type II diabetes ICD9 codes or 2 high A1C lab results) with no history of stroke and who went on to have a stroke within 3 months after administration of glipizide.*

**Table S2.** Query generation, execution time and length for the type II diabetes cohort definition using ACE TQL or BigQuery SQL over a dataset structured using the OMOP CDM v5.3.

|  | ACE TQL | BigQuery SQL, OMOP CDM |
| --- | --- | --- |
| Query generation time | 5 minutes | 150 minutes |
| Query run time | 0.2 seconds | 54.8 seconds |
| Query length | 9 lines | 245 lines |

**Box S1.** The first set of SQL queries necessary to execute the type II diabetes cohort definition using a Google Bigquery instance, with the data structured using the OMOP CDM v5.3, to find male patients.

| CREATE OR REPLACE TABLE  `{work_project_id}.{work_dataset_id}.step1_ace_paper` AS  SELECT  DISTINCT person.person_id  FROM  `{cdm_project_id}.{cdm_dataset_id}.person` person  WHERE  person.gender_concept_id = 8507; |
| --- |

**Box S2**. The second set of SQL queries necessary to execute the type II diabetes cohort definition using a Google Bigquery instance, with the data structured using the OMOP CDM v5.3, to find the first time where patients retrieved by the query in Box S1 had at least 2 subsequent ICD9 250.00 or at least 2 labs 4548-4 [%] with value over 8. At the time of this first mention they had to have been over 65 years old.

| CREATE OR REPLACE TABLE  `{work_project_id}.{work_dataset_id}.step2a_ace_paper` AS  WITH  icd9_codes AS(  SELECT  cr.concept_id_2 AS concept_id  FROM  `{cdm_project_id}.{cdm_dataset_id}.concept` con  JOIN  `{cdm_project_id}.{cdm_dataset_id}.concept_relationship` cr  ON  cr.concept_id_1 = con.concept_id  WHERE  concept_code = '250.50'  AND vocabulary_id = 'ICD9CM'  AND relationship_id = 'Maps to' )  SELECT  cond3.person_id,  cond3.condition_concept_id,  cond3.condition_start_date  FROM (  SELECT  cond2.person_id,  cond2.condition_concept_id,  cond2.condition_start_date,  -- ordering by date so we get the diagnoses per person in sequential order  -- In this way we also get the first mentions  RANK() OVER (PARTITION BY cond2.person_id ORDER BY cond2.condition_start_date ASC ) cond_order  FROM (  SELECT  DISTINCT cond1.person_id,  cond1.condition_concept_id,  cond1.condition_start_date  FROM  `{cdm_project_id}.{cdm_dataset_id}.condition_occurrence` cond1  WHERE  cond1.person_id IN ( -- Restricting to Males  SELECT  person_id  FROM  `{work_project_id}.{work_dataset_id}.step1_ace_paper`)  AND cond1.condition_concept_id IN ( -- Restricting to diabetes  SELECT  concept_id  FROM  icd9_codes) ) AS cond2) cond3  WHERE  cond3.cond_order = 2; -- restricting for at least 2 consecutive occurrences  CREATE OR REPLACE TABLE  `{work_project_id}.{work_dataset_id}.step2b_ace_paper` AS  WITH  loinc_codes AS(  SELECT  cr.concept_id_2 AS concept_id  FROM  `{cdm_project_id}.{cdm_dataset_id}.concept` con  JOIN  `{cdm_project_id}.{cdm_dataset_id}.concept_relationship` cr  ON  cr.concept_id_1 = con.concept_id  WHERE  concept_code = '4548-4'  AND vocabulary_id = 'LOINC'  AND relationship_id = 'Maps to' )  SELECT  meas3.person_id,  meas3.measurement_concept_id,  meas3.measurement_date,  meas3.value_as_number,  meas3.meas_order  FROM (  SELECT  meas2.person_id,  meas2.measurement_concept_id,  meas2.measurement_date,  meas2.value_as_number,  -- Same as before ordering to get the first 2 consecutive occurrences  RANK() OVER (PARTITION BY meas2.person_id ORDER BY meas2.measurement_date ASC ) meas_order  FROM (  SELECT  DISTINCT meas1.person_id,  meas1.measurement_concept_id,  meas1.value_as_number,  meas1.measurement_date  FROM  `{cdm_project_id}.{cdm_dataset_id}.measurement` meas1  WHERE  meas1.person_id IN (  SELECT  person_id  FROM  `{work_project_id}.{work_dataset_id}.step1_ace_paper`)  AND meas1.measurement_concept_id IN (  SELECT  concept_id  FROM  loinc_codes)  AND meas1.value_as_number >= 8) AS meas2) meas3  WHERE  meas3.meas_order = 2;  CREATE OR REPLACE TABLE  `{work_project_id}.{work_dataset_id}.step2_ace_paper` AS  WITH  agg_table AS (  SELECT  person_id,  event_date  FROM ( -- Compute age at event for conditions  SELECT  s2a.person_id,  s2a.condition_start_date AS event_date,  DATE_DIFF(condition_start_date, CAST(p1.birth_DATETIME AS date), YEAR) AS age_at_event  FROM  `{work_project_id}.{work_dataset_id}.step2a_ace_paper` s2a  JOIN  `{cdm_project_id}.{cdm_dataset_id}.person` p1  ON  p1.person_id = s2a.person_id)  WHERE  age_at_event >= 65 -- -- Filter for conditions that happens after 65 years old  UNION ALL  SELECT  person_id,  event_date  FROM ( -- Compute age at event for the measurements  SELECT  s2b.person_id,  s2b.measurement_date AS event_date,  DATE_DIFF(measurement_date, CAST(p1.birth_DATETIME AS date), YEAR) AS age_at_event  FROM  `{work_project_id}.{work_dataset_id}.step2b_ace_paper` s2b  JOIN  `{cdm_project_id}.{cdm_dataset_id}.person` p1  ON  p1.person_id = s2b.person_id)  WHERE  age_at_event >= 65) -- Filter for measurements that happens after 65 years old  SELECT  person_id,  MIN(event_date) AS event_date, -- Select the minimum date since it is an OR for measurement, conditions  FROM  agg_table  GROUP BY  person_id; |
| --- |

**Box S3**. The third set of SQL queries necessary to execute the type II diabetes cohort definition using a Google Bigquery instance, with the data structured using the OMOP CDM v5.3, to find all patients that had an instance of RX 310490 followed by an instance of ICD9 434.91 within 3 months, after the diabetes time intervals returned by the query in Box S2.

| CREATE OR REPLACE TABLE  `{work_project_id}.{work_dataset_id}.step3a_ace_paper` AS  WITH  rxnorm_codes AS(  SELECT  cr.concept_id_2 AS concept_id  FROM  `{cdm_project_id}.{cdm_dataset_id}.concept` con  JOIN  `{cdm_project_id}.{cdm_dataset_id}.concept_relationship` cr  ON  cr.concept_id_1 = con.concept_id  WHERE  concept_code = '310490'  AND vocabulary_id = 'RxNorm'  AND relationship_id = 'Maps to' )  SELECT  DISTINCT  drug.person_id,  drug_exposure_start_DATE AS event_date  FROM  `{cdm_project_id}.{cdm_dataset_id}.drug_exposure` drug  JOIN  `{work_project_id}.{work_dataset_id}.step2_ace_paper` s2  ON  -- This ensures is only matching the patients from prior step  s2.person_id = drug.person_id  WHERE  -- This ensures the drug exposure is after the conditions and measurements  drug.drug_exposure_start_date > s2.event_date  AND drug_concept_id IN (  SELECT  concept_id  FROM  rxnorm_codes);  -- Select the first occurrence of ICD9="434.91" for the set of people from prior step  CREATE OR REPLACE TABLE  `{work_project_id}.{work_dataset_id}.step3b_ace_paper` AS  WITH  icd9_codes AS(  SELECT  cr.concept_id_2 AS concept_id  FROM  `{cdm_project_id}.{cdm_dataset_id}.concept` con  JOIN  `{cdm_project_id}.{cdm_dataset_id}.concept_relationship` cr  ON  cr.concept_id_1 = con.concept_id  WHERE  concept_code = '434.91'  AND vocabulary_id = 'ICD9CM'  AND relationship_id = 'Maps to' )  SELECT  cond.person_id,  -- Select the first occurrence for the subset of people that meet prior criteria  MIN(cond.condition_start_date) AS event_date  FROM  `{cdm_project_id}.{cdm_dataset_id}.condition_occurrence` cond  JOIN  `{work_project_id}.{work_dataset_id}.step3a_ace_paper` s3a  ON  s3a.person_id = cond.person_id  WHERE  cond.condition_concept_id IN (  SELECT  concept_id  FROM  icd9_codes)  GROUP BY  cond.person_id  ;  CREATE OR REPLACE TABLE  `{work_project_id}.{work_dataset_id}.step3_ace_paper` AS  SELECT  DISTINCT  s3a.person_id,  s3a.event_date  FROM  -- Subset for the occurrence of condition or measurement  `{work_project_id}.{work_dataset_id}.step3a_ace_paper` s3a  JOIN  -- First occurrence of ICD9="434.91"  `{work_project_id}.{work_dataset_id}.step3b_ace_paper` s3b  ON  s3b.person_id = s3a.person_id  WHERE  -- This ensures the occurrence of the condition is after the medication  s3b.event_date > s3a.event_date  -- This ensures that ICD9="434.91" occurs within 3 months  AND DATE_DIFF(s3a.event_date, s3b.event_date, MONTH) <= 3 |
| --- |

**Box S4.** The final SQL query necessary to execute the type II diabetes cohort definition using a Google Bigquery instance, with the data structured using the OMOP CDM v5.3, to retrieve the patients and drug exposure start times returned by the queries in Box S3.

| SELECT  *  FROM  `{work_project_id}.{work_dataset_id}.step3_ace_paper` |
| --- |

## ACE licensing details

ACE is freely available for non-commercial use via a complementary license from the Stanford Office of Technology Licensing (https://otl.stanford.edu/) as technology asset S16-392, or via a service contract with Odysseus Data Services (https://odysseusinc.com/).
